# Cell-penetrating peptides enhance peptide vaccine accumulation and persistence in lymph nodes to drive immunogenicity

**DOI:** 10.1073/pnas.2204078119

**Published:** 2022-08-01

**Authors:** Coralie M. Backlund, Rebecca L. Holden, Kelly D. Moynihan, Daniel Garafola, Charlotte Farquhar, Naveen K. Mehta, Laura Maiorino, Sydney Pham, J. Bryan Iorgulescu, David A. Reardon, Catherine J. Wu, Bradley L. Pentelute, Darrell J. Irvine

**Affiliations:** ^a^The Koch Institute for Integrative Cancer Research, Massachusetts Institute of Technology, Cambridge, MA 02142;; ^b^Department of Chemistry, Massachusetts Institute of Technology, Cambridge, MA 02139;; ^c^Department of Biological Engineering, Massachusetts Institute of Technology, Cambridge, MA 02139;; ^d^Department of Pathology, Brigham and Women's Hospital, Harvard Medical School, Boston, MA 02115;; ^e^Department of Medical Oncology, Dana-Farber Cancer Institute, Boston, MA 02215;; ^f^Broad Institute of MIT and Harvard, Cambridge, MA 02142;; ^g^Center for Neuro-Oncology, Dana-Farber Cancer Institute, Harvard University School of Medicine, Boston, MA 02215;; ^h^Center for Environmental Health Sciences, Massachusetts Institute of Technology, Cambridge, MA 02139;; ^i^Ragon Institute of Massachusetts General Hospital, Massachusetts Institute of Technology and Harvard University, Cambridge, MA 02139;; ^j^Department of Materials Science and Engineering, Massachusetts Institute of Technology, Cambridge, MA 02139;; ^k^Howard Hughes Medical Institute, Chevy Chase, MD 20815

**Keywords:** cell penetrating peptides, peptide vaccines, cancer immunotherapy

## Abstract

Peptide-based cancer vaccines are widely investigated in the clinic but exhibit modest immunogenicity. One approach that has been explored to enhance peptide vaccine potency is covalent conjugation of antigens with cell-penetrating peptides (CPPs), linear cationic and amphiphilic peptide sequences designed to promote intracellular delivery of associated cargos. Antigen-CPPs have been reported to exhibit enhanced immunogenicity compared to free peptides, but their mechanisms of action in vivo are poorly understood. We tested eight previously described CPPs conjugated to antigens from multiple syngeneic murine tumor models and found that linkage to CPPs enhanced peptide vaccine potency in vivo by as much as 25-fold. Linkage of antigens to CPPs did not impact dendritic cell activation but did promote uptake of linked antigens by dendritic cells both in vitro and in vivo. However, T cell priming in vivo required *Batf3*-dependent dendritic cells, suggesting that antigens delivered by CPP peptides were predominantly presented via the process of cross-presentation and not through CPP-mediated cytosolic delivery of peptide to the classical MHC class I antigen processing pathway. Unexpectedly, we observed that many CPPs significantly enhanced antigen accumulation in draining lymph nodes. This effect was associated with the ability of CPPs to bind to lymph-trafficking lipoproteins and protection of CPP-antigens from proteolytic degradation in serum. These two effects resulted in prolonged presentation of CPP-peptides in draining lymph nodes, leading to robust T cell priming and expansion. Thus, CPPs can act through multiple unappreciated mechanisms to enhance T cell priming that can be exploited for cancer vaccines with enhanced potency.

## Significance

Current peptide antigen vaccine strategies have shown modest success in eliciting robust T cell priming. We defined mechanisms of action for a promising strategy based on covalently linking peptide antigens to cell-penetrating peptides (CPPs). We found that CPPs enhance CD8^+^ T cell priming through improved lymph trafficking/lymph node accumulation, higher uptake by antigen-presenting cells, increased peptide stability, and prolonged exposure of the antigen in the draining lymph node. Understanding the mechanisms by which CPPs enhance the immune response toward tumor antigens provides a foundation for future applications of peptide vaccines in combination immunotherapies.

Therapeutic peptide vaccines are a promising class of cancer immunotherapy with the potential to initiate, guide, and strengthen an antitumor T cell response (1, 2). Peptide antigens can be manufactured rapidly at reasonable costs, facilitating expedited testing during development and enabling the production of patient-specific neoantigen vaccines (3–5). Often these vaccines comprise “long peptide” amino acid sequences that include more than a minimal CD4^+^ or CD8^+^ T cell epitope to increase the number of possible HLA-peptide epitopes that are contained in the vaccine and to avoid promiscuous presentation of optimal epitopes on nonprofessional antigen-presenting cells (APCs) (6). Peptide vaccines, including neoantigen vaccines, have been demonstrated to prime T cells in patients, but the suboptimal potency of these responses leaves substantial room for improvement (7–11). A variety of approaches are being explored to enhance the response to peptide vaccines, including optimization of adjuvants, dosing intervals, and combining vaccines with immune checkpoint blockade and other immunotherapies (1, 2, 12–18). A complementary strategy is to improve the delivery of the vaccine molecules themselves. This is a particularly relevant problem for peptide vaccines, which exhibit poor uptake into the lymphatics and a short half-life in vivo (19–21). Designing peptide vaccines for efficient delivery has the potential to significantly boost their immunogenicity and consequent antitumor efficacy.

Vaccine antigen delivery is a multistep process that includes both trafficking to the appropriate lymphoid tissues and uptake by key APCs. In order to prime an efficient T cell response, peptide antigens must traffic to lymph nodes (LNs) draining the vaccine injection site. The vaccine may be actively transported by migratory APCs at the injection site or directly enter lymphatic vessels and be passively transported to the draining lymph nodes (dLNs) (19, 20, 21). dLN delivery of unmodified peptide antigens is greatly limited by rapid degradation by peptidases and clearance of these low molar mass compounds into the blood vasculature rather than lymphatic vessels draining the injection site (20, 22). After reaching the LN, vaccine antigens must be internalized by dendritic cells (DCs), proteolytically processed, and presented on MHC molecules to cognate T cells. Uptake of extracellular antigens for presentation on class I MHC to CD8^+^ T cells is a process called cross presentation that is largely restricted to type 1 conventional DCs (cDC1) (23). Some approaches to improve vaccine efficiency have thus used antibodies or other targeting moieties to boost vaccine delivery to these cross-presenting cDC1s (24, 25).

An alternative strategy might be to formulate vaccine antigens that promote direct translocation into the cytosol of APCs, thereby accessing the subcellular compartments where classical MHC class I peptide loading occurs. Such an approach would in principle enable any DC (and not just cross-presentation–competent cells) to present vaccine antigens on class I MHC, increasing the pool of APCs able to prime CD8^+^ T cells in the dLN. This thinking has motivated studies pairing vaccine antigens with cell-penetrating peptides (CPPs) (26, 27). CPPs are linear peptides ∼5- to 40-amino acid residues in length that are generally comprised of cationic or amphipathic membrane-interactive sequences. CPPs can be natural sequences derived from proteins (e.g., penetratin from the Antennapedia homeodomain of *Drosophila*) (28), designed de novo sequences (e.g., Arg_8_) (26, 27), or hybrid natural/synthetic sequences (e.g., Bpep) (29). They can promote endocytosis and, in some cases, direct cytosolic delivery of linked cargos ranging from small molecules to oligonucleotides, and proteins via interactions with various components of the cell surface, including glycans, endocytic receptors, and the plasma membrane itself (30–32). CPPs have been shown to promote DC uptake of antigen peptides in vitro, to enhance vaccine immunogenicity in animal models including nonhuman primates, and to improve the antitumor efficacy of cancer vaccines in mice (33–41); early clinical trials of antigen-CPP vaccines are ongoing (clinicaltrials.gov: NCT04046445). However, many of these studies have examined only a limited set of CPP sequences and have primarily employed highly immunogenic model antigens, such as the ovalbumin epitope SIINFEKL (35–38). More importantly, the means by which CPPs enhance vaccine efficacy remains underexplored. In vitro, conjugation to the potent CPP penetratin (pAntp) has been shown to enhance antigen uptake by DCs, with confocal microscopy suggesting some direct cytosolic delivery and evidence of proteasomal-dependent antigen presentation on class I MHC (42, 43). However, it remains unclear whether similar mechanisms govern the efficacy of CPP conjugates in vivo.

Herein, we sought to compare the immunogenicity of a panel of CPP-antigen conjugates to determine which sequences were particularly effective in boosting T cell responses, and to define mechanisms of action underlying their efficacy in vivo. We found that three of the eight tested CPPs increased polyfunctional CD8^+^ T cell responses to peptide antigens up to 25-fold. Despite evidence from prior studies suggesting that CPPs can deliver antigen to the cytoplasm in vitro, T cell responses to antigen-CPPs in vivo required *Batf3*-dependent cross-presenting DCs. However, CPPs did increase in vivo uptake into LN DCs and other APCs, presumably via endocytosis. Unexpectedly, CPPs also promoted antigen trafficking to LNs, improved antigen stability, and prolonged robust antigen presentation compared to free peptide antigens by at least 2 wk after immunization. Taken together, these factors contributed to substantial enhancements in T cell expansion and functionality.

## Results

### Antigen-CPP Conjugates Enhance T Cell Priming In Vitro and In Vivo.

**Fig. 1.**
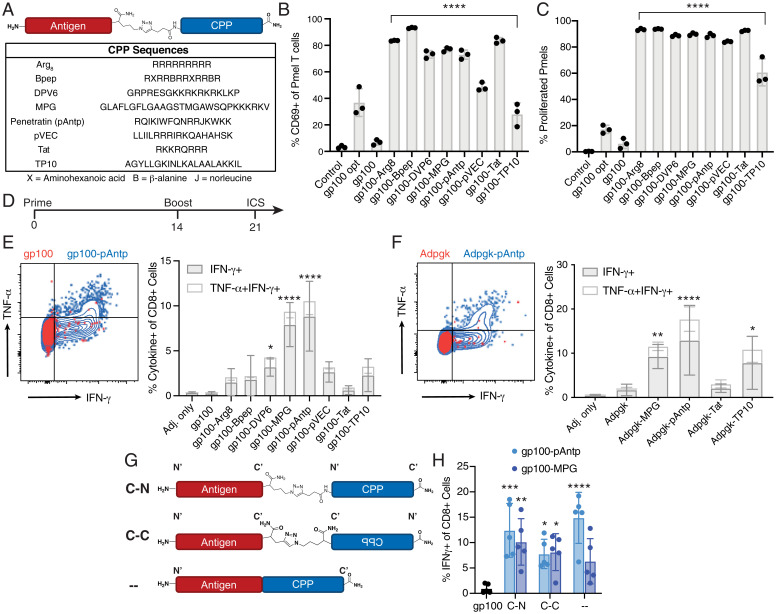
Conjugating peptide antigens to CPPs increases antigen-specific T cell responses in vitro and in vivo. (*A*) Schematic structure of antigen-CPP conjugates prepared using azide/alkyne click chemistry and sequences of eight tested CPPs. (*B* and *C*) Splenocytes from C57BL/6 mice were pulsed for 1 h with the indicated gp100 peptide or CPP conjugate then cocultured with naïve CFSE-labeled pmel-1 CD8^+^ T cells. Pmel-1 T cell activation was assessed by flow cytometry analysis of CD69 up-regulation at 24 h (*B*) and proliferation (CFSE dilution) at 72 h (*C*). Significance relative to the gp100 long peptide was determined by one-way ANOVA followed by Dunnett’s multiple comparisons test: ****P < 0.0001. (*D–H*) C57BL/6 mice (*n* = 4 to 5 animals per group) were immunized twice with 5 nmol of the indicated antigen and 25 µg cyclic-di-GMP, followed by restimulation of peripheral blood mononuclear cells (PBMCs) at day 21 with peptide to detect cytokine-producing antigen-specific T cells by flow cytometry. (*D*) Timeline of immunization experiments. (*E*) Representative flow cytometry plots (*Left*) and quantification (*Right*) of gp100-specific IFN-γ**^+^**/TNF-α**^+^** CD8**^+^** T cells. (*F*) Representative flow cytometry plots (*Left*) and quantification (*Right*) of Adpgk-specific IFN-γ**^+^**/TNF-α**^+^** CD8**^+^** T cells. (*G*) Linker placement for the antigen gp100 to pAntp and MPG. The original, a click linkage between the antigen C terminus and the CPP N terminus, is denoted “c-n”; “c-c” denotes a click linker between the antigen C terminus and the CPP C terminus; “–“ denotes a variant with a peptide bond linking the antigen and the CPP (i.e., synthesizing the antigen-CPP as a single long peptide). (*H*) Percentage of IFN-γ**^+^** of CD8**^+^** T cells after a prime and boost with the CPP conjugates shown in *G*. Significance relative to the gp100 long peptide (or Adpgk) was determined by two-way ANOVA followed by Dunnett’s multiple comparisons test: ****P < 0.0001, ***P < 0.001, **P < 0.01, and *P < 0.05.

To compare the ability of CPPs to enhance the immunogenicity of peptide vaccines, we selected eight previously reported sequences that ranged in their hydrophobicity, net charge, length, and origin. We included several CPPs that have been previously reported to augment peptide vaccines: penetratin (pAntp) (36,39), HIV transactivator of transcription (Tat) (38, 44), Arg_8_ (45), and MPG (46). Additionally, this series included CPPs that, to our knowledge, have never been tested for vaccine antigen delivery: Bpep (29), Diatos peptide vector 6 (DPV6) (47), vascular endothelial cadherin-derived peptide (pVEC) (48), and Transportan 10 (TP10) (49). Each CPP was conjugated via copper(I)-catalyzed click chemistry to the C terminus of an 18-mer-long peptide (gp100_20–38_, hereafter gp100) encompassing the H-2D^b^–restricted epitope EGPRNQDWL (gp100_25–33_) of the melanoma tumor-associated antigen (Fig. 1*A*) (50, 51).

We first assessed the impact of CPP conjugation on antigen presentation in vitro. Murine splenocytes were pulsed with free gp100 peptide, the optimal nonamer epitope, or the gp100-CPP conjugate and then cocultured with naïve 5-(and 6)-Carboxyfluorescein diacetate succinimidyl ester (CFSE)-labeled pmel-1 T cells, which express a transgenic T cell receptor (TCR)-specific for gp100 presented by H-2D^b^ (52). All of the tested CPP conjugates enhanced pmel-1 T cell activation, with up to 13-fold increases in the frequency of T cells up-regulating the early activation marker CD69 and 10- to 15-fold increases in the proportion of T cells proliferating relative to the free gp100_20–38_ antigen alone (Fig. 1 *B* and *C* and *SI Appendix*, Fig. S1). Hence, in line with previous reports, CPP conjugation augmented CD8^+^ T cell priming by peptide antigens in vitro.

We next assessed the potency of antigen-CPP conjugates in vivo. C57BL/6 mice were immunized twice with 10 µg of free gp100 peptide or the molar equivalent of gp100-CPP, each mixed with the STING agonist cyclic di-GMP as an adjuvant (53, 54). Antigen-specific CD8^+^ T cell responses in the peripheral blood were assessed by intracellular cytokine staining for interferon (IFN)-γ and tumor necrosis factor (TNF)-α 1 wk postboost (Fig. 1*D*). As shown in Fig. 1*E*, while all CPPs trended toward enhancing gp100-specific T cell priming in vivo, MPG and pAntp were the most effective, increasing the response ∼25-fold with ∼10% of circulating CD8^+^ T cells reactive to the vaccine antigen. We repeated this immunization study using CPPs conjugated to the neoantigen Adpgk expressed by the MC-38 murine colorectal carcinoma model (55). Similar to the findings with gp100, conjugation to MPG or pAntp enhanced the immunogenicity of the Adpgk peptide (*P* = 0.0027 for MPG, *P* < 0.0001 for pAntp), and these two CPPs elicited the strongest cytokine response among the conjugates tested (Fig. 1*F*).

For these two most-effective CPPs, we explored whether linkage chemistry or construct orientation impacted the T cell response by testing several variations of the antigen-CPP design. MPG and pAntp conjugates were prepared by linking the C terminus of the gp100 peptide to the N or C terminus of the CPP via a triazole linker (i.e., using click chemistry), or by synthesizing the gp100-CPP as a single continuous sequence linked by a standard peptide bond (Fig. 1*G*). While click chemistry was convenient for screening multiple CPP and antigen combinations, peptide bonds—where the antigen-CPP is produced as a single peptide sequence—are more standard and have been used in clinical trials involving CPPs and other synthetic peptides (clinicaltrials.gov: NCT04046445, NCT05098210). All three antigen-CPP designs were substantially more immunogenic relative to free peptide with no significant differences based on linker placement or chemistry (Fig. 1*H*). We also found that scrambling the CPP sequences trended toward improved T cell priming when compared with the antigen alone, but this did not reach statistical significance (*SI Appendix*, Fig. S2). Using ELISpot, an IFN-γ T cell response was detected against the MPG CPP sequence itself after multiple doses, but not pAntp (*SI Appendix*, Fig. S3). However, restimulation of antigen-CPP–primed T cells using mismatched epitope sequences (relative to the vaccine antigen) yielded no detectable response, confirming that the observed immunogenicity is specific and antigen-dependent (*SI Appendix*, Fig. S4). Consistent with prior reports, we found that CPPs are broadly capable of enhancing the immunogenicity of peptide antigens in vivo and that construct design has minimal impact on the immunogenicity. Based on these findings, we focused on conjugates of pAntp connected to peptide antigens via a simple peptide bond for further studies.

### Antigen-CPP Conjugates Elicit Larger Populations of Functional Effector CD8^+^ T Cells.

**Fig. 2.**
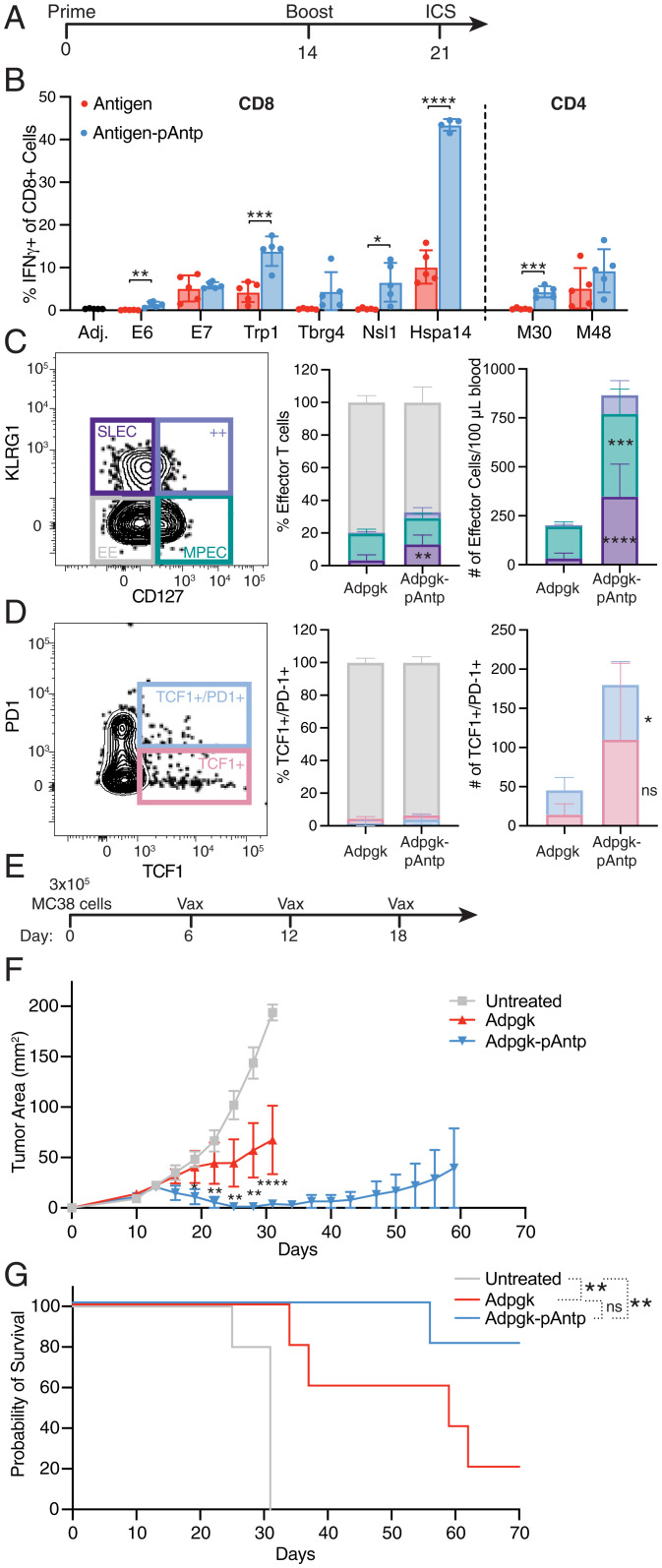
Conjugation of peptide antigens to pAntp enhances vaccine potency without skewing T cell phenotypes, leading to robust expansion of therapeutically effective T cells. (*A*) Timeline of immunization experiments. (*B*) Percentage of IFN-γ**^+^** of CD8**^+^** T cells (or CD4^+^ cells for M30 and M48) after a prime and boost using the indicated long antigen peptide or antigen-pAntp, along with c-di-GMP adjuvant, as compared to the respective antigen. (*C* and *D*) C57BL C57BL/6 mice (*n* = 5 animals per group) were immunized with 5 nmol Adpgk or Adpgk-pAntp peptide combined with 25 µg cyclic-di-GMP on days 0 and 14, and then on day 21 PBMCs were restimulated with optimal Adpgk peptide ex vivo and stained for flow cytometry analysis. Shown are representative flow cytometry plots, percentages, and quantification of: (*C*) CD8^+^/IFN-γ^+^/IL-7R^hi^/KLRG1^lo^ MPECs, CD8^+^/IFN-γ^+^/IL-7R^lo^/KLRG1^hi^ SLECs, and CD8^+^/IFN-γ^+^/IL-7R^lo^/KLRG1^lo^ early effectors (EE); (*D*) PD-1^+^/TCF-1^+^ expression. Statistical analyses for *C* and *D* were performed using a two-way ANOVA with Šídák’s multiple comparisons test. (*E*) Timeline for therapeutic efficacy study using the MC-38 tumor model. (*F*) Corresponding tumor growth curves with a two-way ANOVA statistical comparison followed by Tukey posttest of untreated control to Adgpk-pAntp. (*G*) overall survival curves, analyzed with the Mantel–Cox log-rank test. For all sections ****P < 0.0001; ***P < 0.001; **P < 0.01, *P < 0.05; n.s., not significant.

To assess whether attaching pAntp could be a general strategy for improving peptide vaccines, conjugates of this CPP with antigens from several additional tumor models were synthesized and assessed for immunogenicity following the immunization scheme in Fig. 2*A*. To further explore enhancing an antigen-specific response across antigen classes, pAntp was conjugated to the tumor-associated antigen Trp1 from the immunoresistant B16F10 murine melanoma, to three neoantigens identified from the immunosensitive GL261 and immunoresistant CT2A murine glioma models (Nsl1, Tbrg4, and Hspa14) (*SI Appendix*, Fig. S5), as well as the human papillomavirus oncoviral antigens E6 and E7 (Fig. 2*B*). In addition to these CD8 epitopes, pAntp was also conjugated to two MHC class II-restricted neoantigens from the B16F10 model (M30 and M48) (56) to examine its impact on priming IFN-γ^+^ CD4^+^ T cells (Fig. 2*B*). Altogether, from a set containing tumor-associated, neo-, and oncoviral antigens, pAntp conjugation enhanced responses to five of the eight antigens (with a trend toward increased responses for an additional two of the epitopes), including one of the MHC-II antigens, demonstrating the general utility of this CPP for increasing peptide-specific vaccine responses.

We next assessed the phenotype of the CD8^+^ T cells expanded by antigen-CPP vaccination (57). Free Adpgk peptide and Adpgk-pAntp immunizations elicited similar proportions of interleukin (IL)-7R–expressing memory precursor effector cells (MPECs) (Fig. 2*C*). However, the CPP-conjugate elicited a much greater expansion in the total number of antigen-specific T cells, hence both MPEC and short-lived effector cell (SLEC) T cell populations were present at substantially higher numbers in the blood after CPP vaccination compared to free peptide immunization (*P* = 0.0005 for MPECs, *P* < 0.0001 for SLECs) (Fig. 2*C*). Additionally, the CPP vaccine elicited a similar proportion of both exhausted PD-1^+^/TIM-3^+^ cells and PD-1^+^/TCF-1^+^ stem-like T cells, but the stem-like antigen-specific T cell population was numerically eightfold greater (Fig. 2*D* and *SI Appendix*, Fig. S6).

To verify the functionality of antigen-primed T cells, we subcutaneously implanted MC-38 tumors in the flank of mice and therapeutically vaccinated with soluble Adpgk peptide or Adpgk-pAntp (Fig. 2*E*), along with anti–PD-1 every 3 d. While Adpgk peptide vaccination had a moderate impact on tumor progression and overall survival, the Adpgk-pAntp vaccine regressed tumors in a majority (four of five) of mice and prolonged median survival (Fig. 2 *F* and *G*). Thus, CPP conjugation enabled peptide vaccines to prime a much larger, polyfunctional T cell population without driving cells into an overtly exhausted or dysfunctional state, thereby leading to enhanced antitumor efficacy.

### CPPs Promote Uptake into DCs In Vitro.

**Fig. 3.**
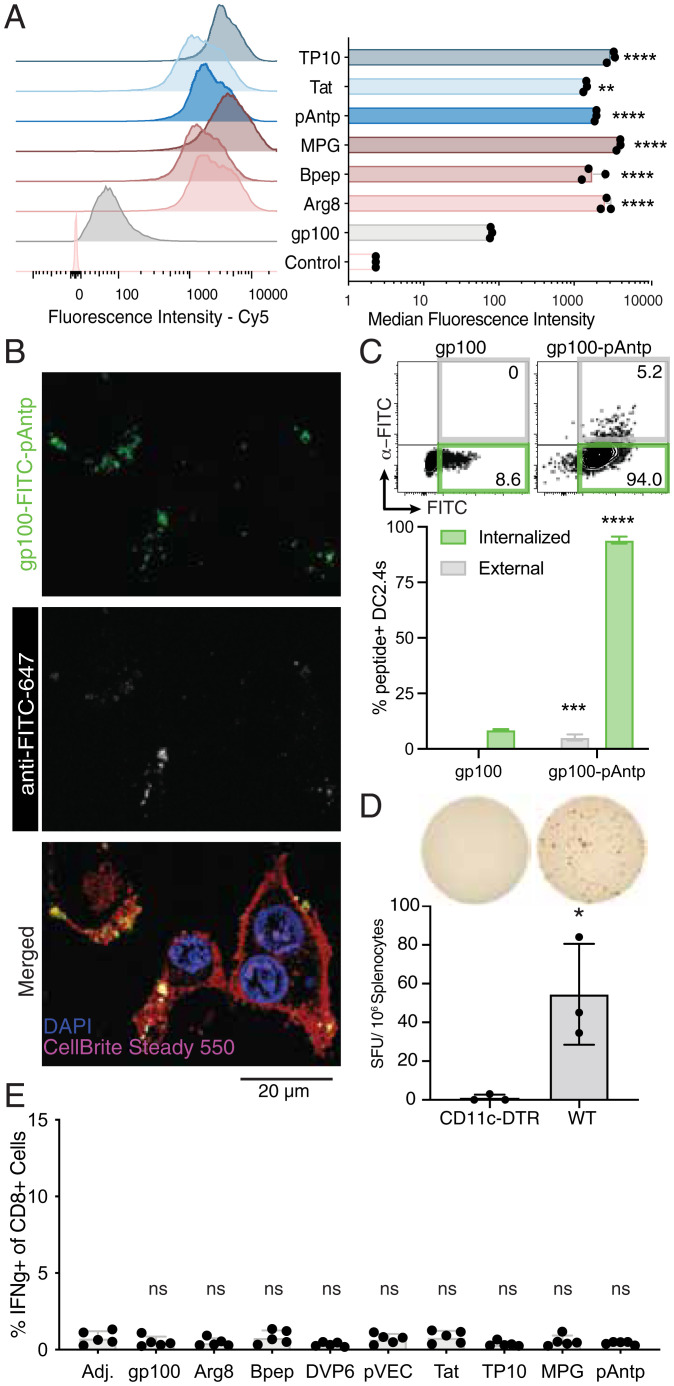
CPPs enhance peptide antigen uptake in vitro but are dependent on cross-presenting cells to promote T cell priming in vivo. (*A*) Representative histograms and median fluorescence intensities of cy5-gp100 and cy5-gp100-CPP in DC2.4 cells after 1-h incubation as determined by flow cytometry. ****P < 0.0001, **P < 0.01 vs. cy5-gp100 determined by one-way ANOVA using Bonferroni’s multiple comparisons test. (*B*) Confocal microscopy of DC2.4 cells stained with a membrane dye (CellBright Steady Membrane 550), Hoechst, and anti-FITC-AF647 to discern membrane-bound and internalized FITC-gp100-pAntp after 1-h incubation with the peptide. (*C*) Percentage of antigen-FITC internalized or associated with the surface of DC2.4 cells after 4 h of incubation with 2.5 µM peptide. Surface-localized antigen was detected using anti–FITC-cy5. ****P < 0.0001, ***P < 0.001 vs. nonfluorescent gp100 control determined by two-way ANOVA with Šídák’s multiple comparisons test. (*D*) C57BL/6 (wild-type, WT) or CD11c-DTR mice (*n* = 3 animals per group) were treated with 550 ng diphtheria toxin followed 24 h later by immunization with 5 nmol gp100-pAntp and c-di-GMP adjuvant. Six days after the priming dose, T cell responses were measured by ELISpot of splenocytes. (*Upper*) Representative images. (*Lower*) Quantification of IFN-γ^+^ spot-forming units (SFUs) *P < 0.05 by Student’s *t* test vs. CD11c-DTR. (*E*) *Batf3^−/−^* mice (*n* = 5 animals per group) were immunized twice with 5 nmol gp100-pAntp and c-di-GMP adjuvant and the percentage of antigen-specific IFN-γ^+^/CD8^+^ T cells was analyzed as in Fig. 2*A*. n.s., not significant by one-way ANOVA followed by Bonferroni posttest vs. adjuvant-only control.

We sought to examine the means by which antigen-CPPs enhance vaccine immunogenicity. We began with one expected mechanism: the boosting of intracellular delivery into APCs, which was based on previous reports demonstrating that CPPs promote cell uptake of covalently linked peptide and protein antigens (45, 58–60). We first compared association with DC in vitro (representing both surface binding and internalization) for the panel of eight CPPs head-to-head. Incubation of DC2.4 DCs with fluorescently labeled gp100 or gp100-CPPs for 1 h revealed a substantial increase in cell-associated antigen for all of the CPP conjugates, ranging from 18-fold with Tat and Bpep to 50-fold with MPG (Fig. 3*A*). Consistent with this finding, confocal imaging revealed substantially higher peptide signal in DCs incubated with gp100-CPPs compared to free gp100 peptide (*SI Appendix*, Fig. S7). Fluorescence of the cell-associated antigen was punctate and exhibited a high degree of colocalization with lysotracker, suggesting that the vaccine peptides were being rapidly internalized and trafficked into the endo-lysosomal pathway. We characterized the extent of internalization for free gp100 vs. gp100-pAntp by incubating DC2.4 cells with FITC-tagged peptides for 4 h, followed by staining with an anti-FITC antibody at 4 °C to detect extracellular antigen. Confocal imaging showed that a majority of gp100-pAntp was already internalized by this time point (Fig. 3*B*). Flow cytometry analysis indicated that addition of the CPP pAntp increased both external membrane association and internalization of the antigen-CPP relative to the antigen alone (*P* < 0.0001 for internalized peptide and *P* = 0.0005 for external, cell-associated peptide), with 90% of antigen^+^ cells showing internalization of the peptide (Fig. 3*C*). Hence, CPPs promote rapid uptake of peptide antigen by APCs in vitro.

### T Cell Priming by Antigen-CPPs Is Dependent on Cross-Presentation In Vivo.

Cross-presentation of extracellular antigens on class I MHC is often restricted to *Batf3*-dependent cDC1s, especially for cell-associated antigens (61). However, CPPs are thought to facilitate direct translocation of attached cargos across cellular membranes (32, 37, 62). If antigen-CPPs promote access to the cytosol, they could theoretically enter the classical MHC class I antigen-processing pathway and be presented by a larger and more diverse population of APCs in dLNs. To determine whether antigen-CPPs circumvent canonical cross-presentation pathways, we first examined the role of DCs broadly in priming this vaccine response. Immunization with gp100-pAntp 24 h after administration of diphtheria toxin to CD11c-diphtheria toxin receptor (DTR) mice—which results in the ablation of all CD11c^+^ DCs—failed to generate a detectable T cell response by IFN-γ ELISpot (Fig. 3*D*). Conversely, gp100-specific T cells were readily detected in control wild-type C57BL/6 that received the same treatment (Fig. 3*D*), suggesting that DCs are critical APCs in the CPP vaccine response. We next immunized *Batf3*-deficient mice with a panel of gp100-CPP conjugates. Similar to the CD11c-DTR mice, no antigen-specific T cell priming was detected in response to any of the CPP conjugates in *Batf3*^−/−^ animals (Fig. 3*E*). Altogether, these data suggest that in vivo, peptide-CPP conjugates require uptake and antigen processing/presentation by DC subsets specialized for cross-presentation, similar to extracellular protein particles or cell-associated antigens (60, 61).

### CPPs Promote Antigen Association with APCs in the dLNs.

**Fig. 4.**
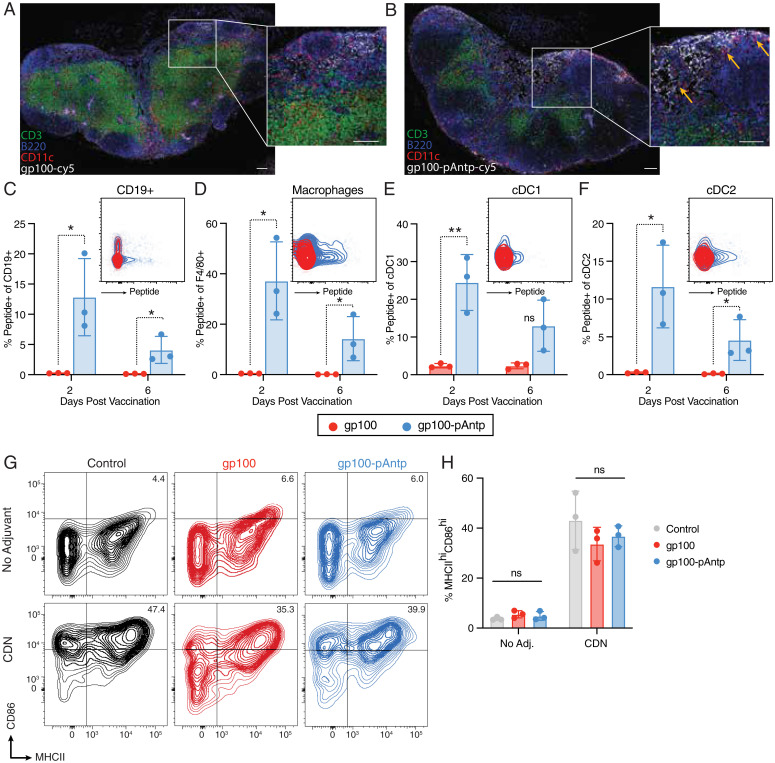
pAntp CPP conjugates exhibit enhanced LN accumulation and uptake by APCs in dLNs relative to free peptide antigen. Groups of C57BL/6 mice (*n* = 3 per group) were immunized subcutaneously with 25 nmol cy5-labeled gp100 or cy5-gp100-pAntp with 25 µg c-di-GMP, and then at selected times inguinal dLNs were isolated for histology or flow cytometry analysis. (*A* and *B*) Immunofluorescence of dLNs 48 h after immunization with cy5-gp100 (*A*) or cy5-gp100-pAntp (*B*). (Scale bars, 100 µm for main images and 50 µm for *Insets*.) (*C–F*) Representative dot plots from day 2 and quantification of percentage of peptide^+^ cells at 2- or 6-d postvaccination with cy5-gp100 and cy5-gp100-pAntp for (*C*) CD19^+^ cells; (*D*) F4/80^+^ macrophages; (*E*) cDC1 cells; and (*F*) cDC2 cells. **P < 0.01, *P < 0.05, by unpaired Student’s *t* test. (*G*) Representative flow cytometry plots showing up-regulation of MHCII and CD86 on CD11c^+^ DCs in response to the vaccination with the gp100 peptides with or without addition of c-di-GMP adjuvant. (*H*) Quantification (*n* = 3 animals per group) of percentage of MHCII^hi^/CD86^hi^ DCs in each condition. n.s., not significant by two-way ANOVA with a Bonferroni posttest.

Although the experiments above suggested that direct cytosolic delivery of antigen is not a key mechanism of antigen-CPP immunogenicity in vivo, CPPs may have multiple other effects on peptide biodistribution and cellular uptake. Therefore, using N terminus-labeled gp100 peptide with or without pAntp conjugation, we investigated the spatiotemporal distribution of peptide within the dLN following immunization. Compared with free peptide (Fig. 4*A*), the addition of pAntp greatly increased the amount of gp100 that accumulated in the dLN and led to deeper penetration of the peptide into the LN paracortex (Fig. 4*B*). The antigen-CPP also exhibited increased colocalization with CD11c^+^ cells at 48 h after injection (Fig. 4 *B*, *Inset* orange arrows). Flow cytometry analysis of LNs following labeled peptide immunization (*SI Appendix*, Fig. S8) showed that addition of pAntp to the antigen facilitated higher association with B cells (Fig. 4*C*), macrophages (Fig. 4*D*), cDC1s (Fig. 4*E*), and cDC2s (Fig. 4*F*), which persisted 6 d following injection. We observed a similar trend with an additional antigen, Tbrg4 (*SI Appendix*, Fig. S9 *A–D*). We next assessed whether the CPP affects APC activation. While the peptides were associated with activated DCs, they did not show a self-adjuvating effect, as up-regulation of CD86 and MHCII on DCs was entirely dependent on the addition of the adjuvant c-di-GMP (Fig. 4 *G* and *H* and *SI Appendix*, Fig. S10).

### CPPs Enhance Trafficking from the Vaccine Site to the dLNs.

**Fig. 5.**
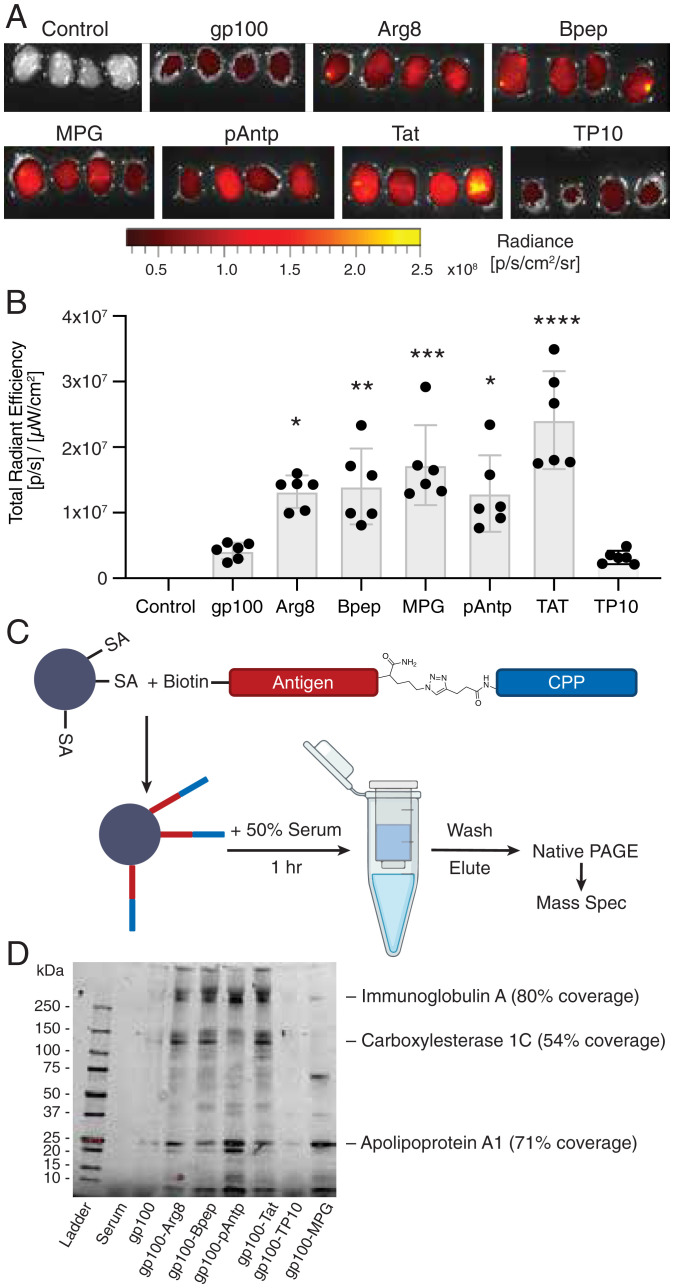
Antigen-CPP conjugates exhibit increased trafficking to dLNs and associate with serum proteins. (*A*) Whole-tissue fluorescence imaging of inguinal LNs 48 h after immunization with 25 nmol-labeled gp100 or gp100-CPPs with 25 µg of cyclic-di-GMP (*n* = 4 LNs per group, image shown at 1.5X)). (*B*) Quantification of total peptide fluorescence from each dLN, compared to control. ****P < 0.0001; ***P < 0.001; **P < 0.01; *P < 0.05; n.s., not significant by one-way ANOVA with Dunnett’s posttest. (*C*) Schematic of antigen pull-down experiment. (*D*) Native-PAGE analysis of proteins pulled down by gp100 or gp100-CPPs following incubation with mouse serum. Several bands identified by LC-MS/MS are indicated.

The observation that pAntp increased antigen accumulation in LN APCs led us to consider the additional mechanisms by which peptide transport to dLNs could be directed by CPPs. For example, low molecular weight peptides are poorly transported into lymphatics following parenteral immunization and are instead predominantly cleared into the blood vasculature, where they are rapidly cleared by the kidneys, whereas higher molecular weight proteins partition efficiently to lymphatic vessels (20, 22). We hypothesized that the physical properties of CPPs might alter peptide trafficking to LNs either by promoting association with cells trafficking into lymphatics from the injection site, by interacting with lymph-trafficking serum proteins, or by both. To quantify changes in total peptide accumulation in dLNs, mice were immunized with fluorescently labeled gp100 peptide or gp100-CPPs and the inguinal LNs were excised 48 h later for whole-tissue fluorescence imaging. All of the tested CPPs, with the exception of TP10, increased accumulation in the inguinal LNs relative to the unconjugated gp100 antigen, ranging from 3.1- to 5.8-fold (Fig. 5 *A* and *B*). We tested the effective CPP pAntp with a second peptide antigen, Tbrg4, and found this antigen-CPP also showed enhanced LN trafficking (*SI Appendix*, Fig. S9 *E* and *F*). None of the CPPs except MPG significantly altered accumulation in the axillary LNs, indicating that CPPs preferentially promoted lymphatic trafficking to the proximal dLNs (*SI Appendix*, Fig. S11).

We postulated that this observed increase in LN accumulation could be due to CPP-mediated binding of the peptides to serum proteins, thereby promoting greater uptake of antigen into lymphatics. To evaluate the interactions between CPPs and serum proteins, we performed a pulldown assay by linking biotinylated gp100-CPPs to streptavidin beads, followed by incubation with serum. After extensive washing, proteins bound to the CPP were eluted using a mild acid and analyzed by native-PAGE (Fig. 5*C*). This experiment revealed minimal interactions of serum proteins with free gp100 peptide (Fig. 5*D* and *SI Appendix*, Fig. S12). In contrast, a set of common protein bands was detected for most of the gp100-CPP conjugates. To identify select proteins enriched in the CPP pulldown, we carried out mass spectrometry (MS) analysis of bands excised from the native-PAGE gels, which identified apolipoprotein A1 as a 30.6-kDa band that prominently bound five of six tested CPPs. Four antigen-CPPs (pAntp, Arg_8_, Bpep, and Tat) also showed association with proteins determined to be IgA and carboxylesterase 1C. Apolipoprotein A1 is the main component in high-density lipoprotein (a naturally occurring nanoparticle) (63), while IgA and carboxylesterase are large proteins with molecular weights of 320 and 180 kDa; these would all be expected to promote size-mediated trafficking of associated CPP-conjugates from the tissue interstitium into lymphatic vessels (64).

### Conjugation to a CPP Improves Antigen Stability in Serum.

**Fig. 6.**
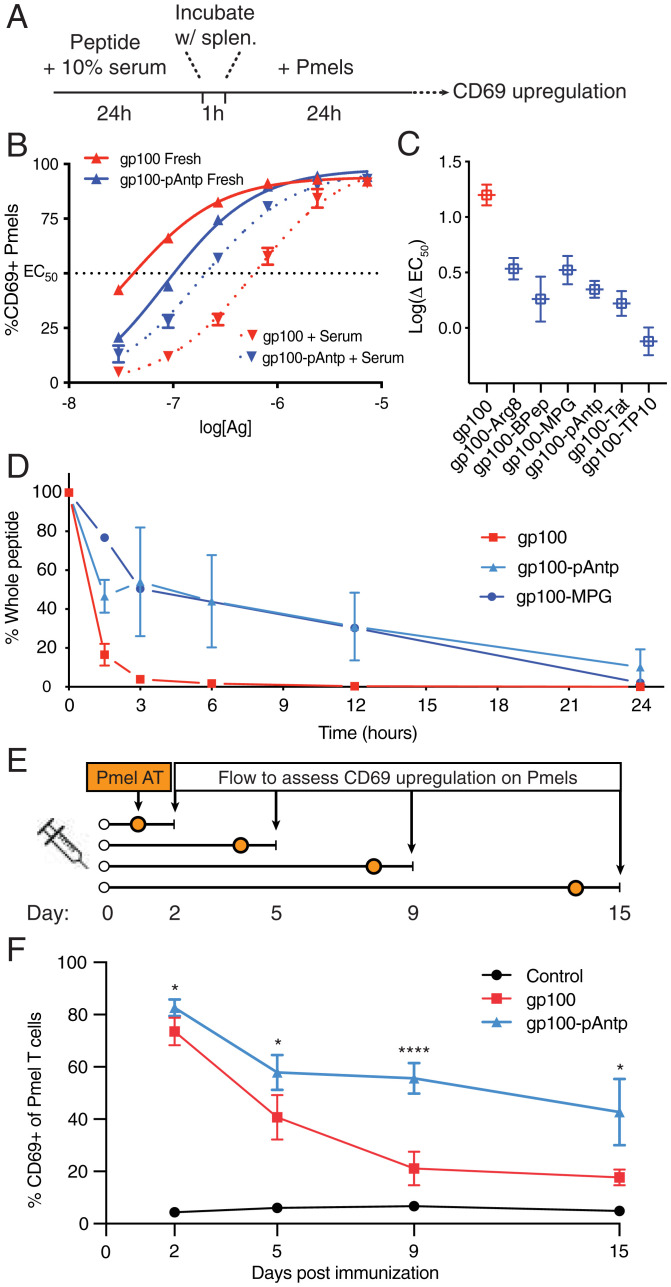
CPPs protect linked antigens from degradation in serum and prolong the duration of antigen presentation in dLNs. (*A*) Timeline of serum exposure T cell activation experiments. (*B*) Representative concentration dependence plots for CD69 up-regulation by pmel-1 T cells cocultured with splenocytes pulsed with the indicated concentration of gp100 or gp100-pAntp peptide with or without preincubation with serum. (*C*) Log fold-change in EC_50_ values for fresh vs. serum-treated peptides for each antigen construct. (*D*) Percentage of intact peptide remaining after incubation in 10% fresh mouse serum as analyzed by LC-QTOF-MS. (*E*) Timeline for experiments assessing the duration of antigen presentation following a single injection of gp100 or gp100-pAntp in C57BL/6 mice. AT, adoptive transfer. (*F*) Levels of available antigen as a function of time postimmunization in dLNs read out by pmel-1 T cell CD69 up-regulation (*n* = 5 animals per group). A two-way ANOVA with a Tukey posttest was used to compare gp100 and gp100-pAntp for each point, with: ****P < 0.0001, **P < 0.01, and *P < 0.05.

Peptides are also highly susceptible to proteolytic cleavage, resulting in a short half-life in vivo (65, 66). We have previously found that peptide antigens designed to bind to the albumin present in interstitial fluid are protected from serum peptidase attack, presumably through steric interference with peptidase recognition (21). To test if the association of antigen-CPPs with serum components impacts peptide antigen stability, we used an in vitro antigen-presentation assay to functionally determine if T cells could still be activated by antigen-CPPs after incubation with serum proteases (22). Free gp100 peptide or gp100-CPP peptides were incubated for 24 h in 10% mouse serum, then added to splenocytes at titrated doses for 1 h to allow antigen uptake and processing, followed by culture with pmel-1 (gp100–specific) T cells for 24 h and assessment of pmel-1 CD69 expression (Fig. 6*A* and *SI Appendix*, Fig. S13). From these cocultures, we determined the peptide antigen concentration required for 50% of maximal CD69 up-regulation by the pmel-1 T cells (EC_50_) (Fig. 6*B*) and the change in EC_50_ for peptides preincubated with serum (ΔEC_50_) (Fig. 6*C*). Incubation of free gp100 peptide in serum led to a substantial loss of pmel-1 stimulation, with an accompanying ∼16-fold increase in EC_50_ (Fig. 6 *B* and *C*). In contrast, the ΔEC_50_ for gp100-CPPs ranged from 0.76 to 3.4 (Fig. 6*C*).

To gain more direct evidence of altered peptide stability, we used quadrupole time-of-flight liquid chromatography/MS (LC-QTOF-MS), to directly detect proteolytic degradation of pAntp and MPG conjugates in serum. Both of these antigen-CPPs exhibited a prolonged lifetime compared to the free gp100 peptide in serum (Fig. 6*D*). We hypothesized that enhanced stability of the conjugates could reflect an intrinsic protective peptidase-/protease-blocking effect by the CPP or be an indirect effect mediated by binding of the conjugate to other serum proteins that sterically interfere with protease attack on the peptide. To distinguish these possibilities, we performed a similar experiment assessing proteolytic stability in the presence of purified proteinase K—a promiscuous protease that cleaves peptide bonds following hydrophobic amino acids—in the absence of other serum components. Interestingly, CPP conjugation did not protect gp100 peptide from rapid degradation in the presence of protease alone (*SI Appendix*, Fig. S14). This suggests that the enhanced antigen stability is indirectly promoted through CPP-serum protein interactions. Overall, the CPPs that were most potent in enhancing the immunogenicity of peptide antigens in vivo also increased the lifetime of linked antigens in the presence of serum.

### CPPs Extend the Duration of Antigen Presentation In Vivo.

Given the findings that linkage to CPPs both increased peptide stability and enhanced accumulation of antigens in dLNs, we assessed the effect of pAntp on the duration of antigen presentation in vivo. C57BL/6 mice were vaccinated with gp100 or gp100-pAntp, and pmel-1 T cells were adoptively transferred 24 h before harvesting the dLNs to serve as reporters of antigen presentation (Fig. 6*E*). Two days after immunization, the activation of pmel-1 T cells was similar between gp100– and gp100-pAntp–vaccinated mice (Fig. 6*F*). However, transfer of reporter pmel-1 cells at later time points revealed a substantial decay in antigen presentation by day 9 following free peptide immunization, while T cells transferred 2 wk postimmunization with pAntp-linked peptide were still robustly activated (Fig. 6*F*). Antigen presentation was also prolonged by the CPP conjugate in the axillary LNs, though to a lesser degree (*SI Appendix*, Fig. S15). Thus, CPP conjugation facilitates robust, extended vaccine antigen presentation in the dLNs.

## Discussion

CPPs have been studied as delivery agents for a range of therapeutics for more than three decades, including phase II clinical trials for the delivery of antisense oligonucleotides (clincialtrials.gov: NCT01396239), and their mechanisms of action have been characterized extensively in vitro. However, their activity in vivo is far less understood. As vaccine carriers, a few CPPs have been shown to improve the immunogenicity of various peptide antigens in both prophylactic and therapeutic immunizations, but mechanisms underlying these enhancements have not been characterized in vivo (45, 58, 59). With this study we aimed to evaluate the generalizability of CPPs as an approach for cancer vaccine delivery and to characterize mechanisms underlying their functionality.

CPP conjugation enhanced the immunogenicity of tumor-associated antigens, neoantigens, and viral antigens, including both MHC class I and II epitopes. CPPs are therefore a general strategy to prime both CD4^+^ and CD8^+^ T cell responses against diverse antigens. We tested 10 different peptide antigens here, representing tumor-associated antigens, oncoviral antigens, and neoantigens. Nine of the 10 peptides tested showed at least trends toward enhanced T cell priming as a CPP conjugate, with the one exception being the immunodominant viral E7 eptiope. It is possible this peptide is an outlier, as it has previously been reported to undergo aggregation with anionic Toll-like receptor agonist adjuvants in a manner impacting immunogenicity (67, 68). In addition, eliciting a successful T cell response requires several steps that the CPP would not be expected to improve, namely proteolytic processing, MHC presentation, and TCR binding. A peptide sequence that does not efficiently undergo these steps would therefore not be expected to exhibit improved immunogenicity with a CPP. Examining the quality of the antigen-specific CD8^+^ T cell responses primed by these vaccines revealed that antigen-CPP conjugates greatly expanded the antigen-specific CD8^+^ T cell population, without strongly skewing the proportions of memory precursor phenotype cells, stem-like cells, or exhausted cells. Thus, the number of antigen-specific MPECs and stem-like T cells, as well as effector cells, was substantially increased by the use of CPP conjugate vaccines. While these findings are likely influenced by the choice of adjuvant, they indicate that CPPs can enhance peptide vaccine potency without necessarily inducing an exhausted or memory-depleted tumor-specific T cell population. Consistent with these findings, antigen-CPP vaccination exhibited enhanced efficacy in mediating therapeutic tumor control.

The current understanding of CPP mechanisms of action would suggest that enhanced T cell priming elicited by antigen-CPPs is mediated by increased intracellular delivery in APCs, either via increased endocytosis or direct translocation across the cell membrane (30–32). We were particularly curious as to whether antigen-CPPs could access the cytosol directly via membrane translocation in vivo, as it would feed directly into the classical MHC-I presentation pathway, allowing antigen presentation by a wider range of APCs. However, neither CD11c-DTR mice (lacking DCs broadly) nor *Batf3* knockout mice (lacking cross-presenting cDC1 cells) (69, 70) were able to mount any antigen-specific CD8^+^ T cell response to the CPP conjugate vaccine. These data suggest that peptide antigen-CPPs are dependent on cross-presentation and cannot directly translocate across the cell membrane. While CPPs do appear to enhance antigen uptake into DCs via endocytosis in vitro, we suspected that alternative mechanisms were responsible for their significant impact on the immune response in vivo.

Turning to explore additional mechanisms of action, we postulated that the amphiphilic nature of CPPs may impart biophysical properties that benefit the peptide antigen. The hydrophobicity and charge imbued by the CPP might act in a similar manner to lipid-based “albumin-hitchhiking” vaccines, which enhance the passive lymphatic trafficking of peptides by binding to serum albumin and increasing the vaccine’s effective molecular weight (16, 20, 21, 71). Whole-LN imaging confirmed that antigen-CPPs accumulated more efficiently in the draining nodes than the free peptide antigen, indicative of CPP-mediated lymphatic trafficking. In vitro analysis of CPP-peptides incubated with serum revealed the binding of antigen-CPPs with apolipoprotein A1, a key component of the high-density lipoprotein complexes that transport fatty acids in the lymph. Interestingly, these naturally occurring nanoparticles (diameters ranging from 7 to 13 nm) fall easily into the optimal nanoparticle size range (<30 nm) for LN trafficking and retention (72, 73). Conjugation to CPPs also enhanced the in vitro stability of a peptide antigen in serum, observed indirectly with a T cell activation assay and directly via LC/MS. Interestingly, this protection was absent when a single isolated protease was incubated with the antigen-CPPs, suggesting that enhanced protection from serum proteases is likely afforded by CPP-mediated association with other serum components (i.e., by sterically hindering access to cleavage sites).

The combined effects of improved antigen stability, enhanced LN accumulation, and efficient uptake by APCs all contribute to prolonged antigen presentation following CPP-peptide immunization. This durable and prolonged presentation in dLNs represents an important integrated mechanism by which CPPs improve CD8^+^ T cell responses to vaccine antigens, consistent with many other preclinical and clinical studies linking prolonged antigen presentation with enhanced CD8^+^ T cell responses to peptide vaccines (16, 20, 74). For example, peptide immunization using the oil-in-water emulsion Montanide combined with a Toll-like receptor 1/2 agonist also leads to prolonged antigen availability and has been recently shown to prime strong CD4^+^ and CD8^+^ T cell responses in human volunteers against SARS-CoV-2 epitopes (75, 76). An intriguing prospect could be to consider combining CPP delivery with such an approach, to further amplify T cell priming via complementary mechanisms of action.

In summary, we have demonstrated the generalizability of CPPs as a strategy to improve T cell responses to peptide vaccines and we defined multiple mechanisms of action underlying antigen-CPP conjugate potency in vivo. While it is likely that further engineering can be undertaken to identify even more effective CPPs, these results provide a foundation for future studies and rationale for the clinical translation of this approach to enhancing peptide vaccines.

## Materials and Methods

### Cells.

DC2.4 cells were provided by K. Rock, University of Massachusetts Medical School, Worcester, MA, and were cultured in complete RPMI-1640 medium (GE Healthcare Life Sciences; supplemented with 10% fetal bovine serum, 100 units/mL penicillin, and 100 μg/mL streptomycin). MC-38 cells were cultured in complete DMEM (GE Healthcare Life Sciences; supplemented with 10% fetal bovine serum, 100 units/mL penicillin, and 100 μg/mL streptomycin). T cells and splenocytes were cultured in complete RPMI with 20 mM Hepes, 1 mM sodium pyruvate, 0.05 mM β-mercaptoethanol, and 1× nonessential amino acids. All cells were maintained at 37 °C and 5% CO_2_.

### Mice.

B6 mice (C57BL/6NTac) were purchased from Taconic. *Batf3*^−/−^ knockout mice [B6.129S(C)-*Batf3^tm1Kmm^*/J] and transgenic pmel-1 mice [B6.Cg-*Thy1^a^*/Cy Tg(TcraTcrb)8Rest/J] were acquired from Jackson Laboratories. Female mice were used between 6 and 8 wk of age unless otherwise noted. All animal work was conducted under the approval of the Massachusetts Institute of Technology Division of Comparative Medicine in accordance with federal, state, and local guidelines.

### Peptides.

Peptides were synthesized using a custom-built rapid-flow automated peptide synthesizer as previously described (77). Briefly, Fmoc-protected amino acids were iteratively attached to a RINK-amide resin support with coupling reactions comprising 0.2 M amino acid, 0.19 M of an activating agent, and 5% (vol/vol) diisopropylethylamine in N,*N*-dimethylformamide (DMF), followed by Fmoc-removal with piperidine (20% vol/vol) in DMF, with DMF washes after each step. All steps were performed at 90 °C and with a flow rate of 80 mL/min. Synthesized peptides were then cleaved from the resin support and purified using reversed-phase high-performance LC. Further experimental detail is provided in *SI Appendix*. For gp100 experiments, the EGPRNQDWL modification of the optimal epitope was used.

### In Vitro T Cell Activation Assay.

Splenocytes were harvested from wild-type C57BL/6 mice and plated with 10^5^ cells per well in a 96-well plate. Splenocytes were treated for 1 h with 0.5 µM of indicated gp100 peptides, then fresh media was exchanged and splenocytes were cocultured with 5 × 10^4^ CFSE-stained pmel-1 T cells. Activation of the pmel-1 T cells was measured at 24 h by flow cytometry via staining with antibodies against Thy1.1 and CD69. Pmel-1 proliferation as determined by CFSE dilution was assessed at 72 h by flow cytometry.

### Prophylactic Vaccination.

Wild-type C57BL/6 and *Batf3*^−/−^ mice were primed with 5 nmol of peptide vaccine and 25 µg c-di-GMP subcutaneously, with half of the dose given on each side of the tail base, followed by a boost of the same strategy 14 d later. For some experiments, a second boost vaccine was given at 21 d after the initial prime. Peripheral blood was analyzed 7 d after the boost by intracellular cytokine staining to detect IFN-γ and TNF-α. Briefly, peripheral blood was collected, red blood cells were lysed, and lymphocytes were plated with 10 µg/mL of the peptide antigen’s optimal epitope. After 2 h of incubation at 37 °C and 5% CO_2_, brefeldin A was added and samples were returned to incubation for an additional 4 h. Samples were stained with a fixable viability dye followed by extracellular staining. Using a fixation/permeabilization kit (BD Biosciences), samples were prepared for intracellular antibody staining and assessed by flow cytometry.

### Tumor Inoculation and Therapy.

For tumor inoculation, 300,000 MC-38 tumor cells were injected subcutaneously on the upper right flank of C57BL/6 mice. Mice were randomized on day 10 with an average tumor size of ∼20 mm^2^, then vaccinated with 5 nmol of peptide vaccine and 25 µg c-di-GMP subcutaneously at the tail base. Subsequent vaccinations were performed on days 16 and 22. Peripheral blood was analyzed 6 d after each vaccination by intracellular cytokine staining. Tumor size was measured as area (longest dimension × perpendicular dimension) every 3 d.

### DC2.4 Uptake Assay and Confocal Imaging.

For confocal analysis, DC2.4 cells were seeded for 48 h in eight-well μ-slides (Ibidi) prior to treatment with 2.5 µM fluor-peptide (4 h), then extensively washed with phosphate-buffered saline (PBS) to remove surface-bound peptide and fluorophores. Samples were stained with Hoechst 33342 Solution (BD Biosciences) and CellBright Steady Membrane 550 (Biotim) in complete RPMI Lysotracker Green DND-26 (Invitrogen). Cells were then washed and imaged in Live Cell Imaging Solution (Invitrogen). Images of DC2.4 cell lines were acquired using an Olympus Fluoview FV1200 microscope equipped with a 100× objective, and optimum lasers and filter sets. The images within each dataset were acquired under identical settings and subsequently processed using Fiji image analysis software.

For analysis by flow cytometry, 10^5^ cells were seeded 24 h prior to treatment in a 24-well plate. Samples were treated with 2.5 µM cy5-gp100-CPP for 1 h, then extensively washed with PBS and trypsinized, stained with live/dead fixable aqua (BioLegend), and analyzed by flow cytometry.

To image and quantify surface-associated peptide compared with internalized peptide, DC2.4 cells were incubated with 2.5 µM FITC-gp100-pAntp or FITC-gp100 for 4 h then scraped and washed with PBS to remove free-floating peptide. Cells were stained with live/dead fixable aqua (BioLegend) and analyzed by flow cytometry.

### Immunofluorescence Staining.

For LN tissue section imaging, mice were immunized with 25 nmol gp100-CPP-cy5 and 25 µg of c-di-GMP. The inguinal LNs were harvested 48 h postimmunization and fixed with 4% PFA at 4 °C for 18 h. Next, LNs were washed in PBS and embedded in 3% (wt/vol) low-melting agarose at 37 °C. The agarose was allowed to solidify on ice for 15 min before further processing on a vibratome (Leica VT1000S). The 100-μm LN sections were obtained and further processed for immunofluorescence staining. Tissue sections were blocked with 5% donkey serum and 2% bovine serum albumin in PBS for 1 h at room temperature. Staining with primary antibodies (1:100) was performed overnight at 4 °C in blocking buffer using Alexa Fluor 594 anti-mouse CD3 Antibody (BioLegend, #100240), Brilliant violet 421 anti-mouse/human CD45R/B220 Antibody (BioLegend, #103240), and unconjugated anti-CD11c antibody (Cell Signaling, Rabbit mAb 97585S). After 3× washes with PBS, the sections were incubated with secondary antibody in blocking buffer for 1 h in the dark at room temperature (donkey anti-rabbit antibody, DyLight 488- Thermo Fisher Scientific, #SA5-10038). After three washes with PBS, the sections were mounted onto glass slides using mounting media (ProLong Diamond Antifade Mountant, Thermo Fisher Scientific). Images of LNs were then acquired using Leica SP8 laser-scanning confocal microscope with a 25× objective.

### LN Trafficking.

Mice were immunized with 25 nmol fluor-peptide and 25 µg c-di-GMP subcutaneously at the tail base, with half of the dose given at each side, then axillary and inguinal LNs were resected 24 h later. Whole LNs were imaged using an IVIS Spectrum In Vivo Imaging System (Perkin-Elmer) to measure epifluorescence. Radiant efficiency was quantified using Living Image software.

### Serum Protein Pulldown.

Protein binding to the CPPs was assessed using the Pierce Biotinylated Protein Interaction Kit (Thermo Scientific). Briefly, biotinylated CPPs were bound to the provided streptavidin beads. Excess peptide was washed from the column by centrifugation. Fifty percent mouse serum in PBS was applied to the column and incubated at 4 °C for 1 h. The serum proteins were washed off the column using PBS and centrifugation until elute produced undetectable levels of protein as assessed by protein gel electrophoresis. Proteins bound to the CPP-beads were then eluted using the mild acid elution buffer provided with the kit and analyzed by native-PAGE.

Protein bands of interest were excised from the gel and washed with 50% (vol/vol) acetonitrile in 100 mM ammonium bicarbonate, with solution removal after washing. Bands were treated with 10 mM DTT in 50 mM ammonium bicarbonate for 1 h at 56 °C then 55 mM iodoacetamide in 50 mM ammonium bicarbonate for 30 min at room temperature in the dark to reduce and alkylate cysteine residues. Bands were then washed with 50% (vol/vol) acetonitrile in 100 mM ammonium bicarbonate, dried under vacuum, and stored at −20 °C for approximately 2 wk.

The lyophilized bands were then rehydrated in ∼20 µL (sufficient to cover the gel fragments) of a digestion solution comprising 50 mM ammonium bicarbonate, 5 mM CaCl_2_, and 5 ng/μL trypsin, and incubated overnight in a 37 °C water bath. The supernatant was then collected and remaining digested peptides were extracted by treating the gel fragments with 50% acetonitrile and 0.1% formic acid (vol/vol) in ultrapure water, then 80% acetonitrile and 0.1% formic acid (vol/vol) in ultrapure water for 15 min each. The supernatants were collected at each step and pooled, then flash frozen, lyophilized, resuspended in 10% acetonitrile and 0.1% formic acid in ultrapure water (vol/vol), desalted with a C18 zip-tip and analyzed via LC-MS/MS, as previously described (78).

### T Cell Activation-Based Serum Stability Assay.

Following prime and boost of mice with gp100 peptides, CD8^+^ T cell responses were confirmed on day −1. On the day of the experiment, spleens from vaccinated animals were excised, lysed in ACK buffer, pooled, and plated in 96-well plates. Twenty-four hours before ex vivo stimulation, mouse serum from naïve animals was freshly collected in collection tubes with Z-Gel (Sarstedt) to remove clotting factors and used to prepare RPMI-1640 + 10% mouse serum media. Next, 30-μM antigen solutions were prepared in RPMI-1640 + 10% mouse serum and incubated at 37 °C. After a 24-h incubation, fresh antigen was similarly prepared at 30 μM in RPMI-1640 + 10% mouse serum and both solutions were immediately diluted 4× with RPMI-1640 + 10% fetal bovine serum; serial dilutions were prepared and used to restimulate the aforementioned splenocytes from vaccinated animals. CD69^+^/CD8^+^ T cell responses were measured by flow cytometry.

### Pmel-1 In Vivo Activation Assay.

Wild-type mice were injected subcutaneously at the tail base with 25 µg c-di-GMP along with 5 nmol of either gp100 or gp100-pAntp in 100 µL of 75 mM MgCl_2,_ 50 µL per side on day 1, 6, 10, or 13 (one group per time point). On day 14, 10^6^ pmel-1 T cells were injected retro-orbitally. Twenty-four hours later, the axillary and inguinal LNs, along with the spleen, were harvested and stained with the congenic marker Thy1.1, CD69, CD8, CD3, and zombie fixable viability dye. Expression of CD69 on pmel-1 T cells in each tissue was assessed by flow cytometry.

### Antibodies for Flow Cytometry.

CD8a (BioLegend #100712), IFN-γ (BioLegend #505808), TNF-α (BioLegend #506313), CD3e (BioLegend #100222), CD19 (BioLegend #115529), F4/80 (BD Biosciences #565614), CD11b (BioLegend #101243), CD11c (BioLegend #117334), CD169 (BioLegend #142412), CD69 (BioLegend #104508), CD127 (BioLegend #135027), KLRG1 (BioLegend #138427), TCF1 (R&D Systems #IC8224G), PD-1 (BioLegend #135210), TIM-3 (BioLegend #119721), XCR1 (BioLegend #148216), CD172a (BD Biosciences #742205), and Thy1.1 (BioLegend #202524). Viability was assessed by live/dead fixable aqua (BioLegend). All flow cytometry was conducted on either an LSR Fortessa or Symphony (BD Biosciences), data were collected in Diva (BD Biosciences) and analyzed using FlowJo (BD Biosciences).

### Statistical Analysis.

Experiments were not performed in a blinded fashion. Sample sizes were chosen based on estimates from pilot experiments and previously published results, such that the respective statistical tests were appropriately powered. Data were analyzed using GraphPad Prism software and reported as mean ± SEM unless otherwise noted. Survival curves were analyzed using the log-rank (Mantel–Cox) test.

## Supplementary Material

Supplementary File

## Data Availability

All study data are included in the main text and *SI Appendix*.
