# Supplementary material for: Cell-penetrating peptides enhance peptide vaccine accumulation and persistence in lymph nodes to drive immunogenicity
Source: Proc Natl Acad Sci U S A. 2022 Aug 1;119(32):e2204078119. doi: 10.1073/pnas.2204078119 (PMC9371699; doi:10.1073/pnas.2204078119)

Supplementary Information for

Cell penetrating peptides enhance peptide vaccine accumulation and persistence in lymph nodes to drive immunogenicity

Coralie M. Backlund[1‡], Rebecca L. Holden[2‡], Kelly D. Moynihan[1,3], Daniel Garafola[1], Charlotte Farquhar[2], Naveen K. Mehta[1,3], Laura Maiorino[1], Sydney Pham[1], J. Bryan Iorgulescu[4,5,6], David A. Reardon[7], Catherine J. Wu[5,6], Bradley L. Pentelute[1,2,6,8], Darrell J. Irvine[1,3,6,9,10,11]*

Affiliations:

[1]The Koch Institute for Integrative Cancer Research, Massachusetts Institute of Technology, Cambridge, MA 02142, USA
[2]Department of Chemistry, Massachusetts Institute of Technology, Cambridge, MA 02139, USA
[3]Department of Biological Engineering, Massachusetts Institute of Technology, Cambridge, MA 02139, USA
[4]Department of Pathology, Brigham and Women's Hospital, Harvard Medical School, Boston, MA 02115, USA
[5]Department of Medical Oncology, Dana-Farber Cancer Institute, Boston, MA 02215, USA
[6]Broad Institute of MIT and Harvard, Cambridge, MA 02142, USA
[7] Center for Neuro-Oncology, Dana-Farber Cancer Institute and Harvard University School of Medicine, Boston, MA 02215
[8]Center for Environmental Health Sciences, Massachusetts Institute of Technology, Cambridge, MA 02139, USA
[9]Ragon Institute of Massachusetts General Hospital, Massachusetts Institute of Technology and Harvard University, Cambridge, MA 02139, USA
[10]Department of Materials Science and Engineering, Massachusetts Institute of Technology, Cambridge, MA 02139, USA
[11]Howard Hughes Medical Institute, Chevy Chase, MD 20815, USA

‡Indicates equal contributions
*Corresponding author: Darrell J. Irvine
Email: djirvine@mit.edu

This PDF file includes:

Supplementary Materials and Methods
Table S1
Figures S1 to S15
Appendix

Supplementary Materials and Methods

Reagents. H-Rink Amide-ChemMatrix and HMPB-ChemMatrix resins were obtained from PCAS BioMatrix Inc. (St-Jean-sur-Richelieu, Quebec, Canada). 1-[Bis(dimethylamino)methylene]-1H-1,2,3-triazolo[4,5-b]pyridinium-3-oxid-hexafluorophosphate (HATU), 4-pentynoic acid and Fmoc-(azido)ornithine-OH were purchased from Chem-Impex International (Wood Dale, IL). Other Fmoc-protected amino acids were purchased from EMD Millipore (Burlington, MA). 7-Azabenzotriazol-1-yloxy)trispyrrolidinophosphonium hexafluorophosphate (PyAOP) was purchased from P3 BioSystems (Louisville, KY). Peptide synthesis-grade N,N-dimethylformamide (DMF), CH_2CL_2, diethyl ether, and HPLC-grade acetonitrile were obtained from VWR International (Radnor, PA). Sulfo-Cyanine5-maleimide, FITC, and BODIPY-Texas Red-maleimide (BODIPY-TR-maleimide) were purchased from LumiProbe. All other reagents involved in peptide preparation were purchased from Sigma-Aldrich (St. Louis, MO). Milli-Q water was used exclusively.

Peptide synthesis. The antigen peptides and the CPPs included in this manuscript were produced using an automated solid-phase peptide synthesizer as previously described (1). For sequences conjugated using copper(I)-catalyzed azide/alkyne cycloaddition (CuAAC), standard batch couplings were used to couple azido-ornithine at the C-terminus of the antigen peptides and 4-pentynoic acid at the N-terminus of each CPP, with the exception of the ‘C-C’ design variants of pAntp and MPG, in which propargyl glycine was coupled at the C-terminus (1). After synthesis was complete, peptidyl resins were washed with dichloromethane and dried under vacuum. Peptides were cleaved from resin by incubating in 5% water, 5% thioanisole, 5% phenol, and 2.5% ethane dithiol in trifluoroacetic acid (TFA, v/v). Peptides were triturated in cold diethyl ether, resuspended in 50:50 water: acetonitrile (v/v) with 0.1% TFA as an additive, and lyophilized.

Peptide purification. Crude peptides (~200 mg) were suspended in 20 mL of either 5% or 15% acetonitrile in water (v/v) with 0.1% TFA as an additive and filtered through a 0.22 µm syringe filter. Peptides were then purified via preparative mass-directed reversed-phase HPLC (MD RP-HPLC) using water with 0.1% TFA as mobile phase A and acetonitrile with 0.1% TFA as mobile phase B. For each purification, solvent was flowed at 20 mL/min over an Agilent Zorbax 300SB-C3 column (21.2 x 250 mm, 7 µm) using a linear gradient ramping from either 5 to 35% B or 15 to 45% B over 60 minutes, with fractions collected in 10 mL increments. Fractions were pooled according to data from the instrument’s in-line quadrupole mass spectrometer and lyophilized.

Antigen-CPP click conjugates and fluorophore-labeled conjugates were purified in a similar manner post-reaction using MD RP-HPLC. Solvent was flowed at 4 mL/min over an Agilent Zorbax 300SB-C3 column (9.4 x 250 mm, 5 µm) using a linear gradient ramping from either 5 to 35% B or 15 to 45% B over 60 minutes, with fractions collected in 4 mL increments. Fractions were pooled and analyzed as above. All peptides and peptide conjugates were characterized via LC-MS to verify identity and purity.

Antigen-CPP conjugation. The indicated azide-containing long antigens were conjugated to each alkyne-CPP via Cu(I)-catalyzed click chemistry. Approximately 1 µmol of each reagent was dissolved in 30:70 water: dimethyl sulfoxide (DMSO, v/v) that had been sparged with N_2. Reactions were incubated for 1 hour on a nutating mixer, then quenched with the addition of 10 mL water with 0.1% TFA additive. Conjugates were then purified via reversed-phase HPLC as described above and isolated with >90% purity. Product identity and purity were verified via LC-MS.

Fluorophore conjugation. Antigen or antigen-azide peptides (20 mg) were massed out into a 50 mL conical vial and dissolved in 45 mL water, then combined with equimolar BDP-TR-maleimide, FITC, or SulfoCy5-maleimide (50 mM stock in DMSO). Reactions were incubated for 30 minutes at room temperature on a nutating mixer, then filtered with a 0.22 µm syringe filter and purified by RP-HPLC as described above. Alternately, for the antigen-azide peptides, which were subsequently conjugated to various alkyne-CPPs and purified, the reaction mixture was lyophilized immediately without purification in order to minimize loss of material. Product identity and purity were verified via LC-MS.

LC-MS analysis. Unless otherwise stated, LC-MS analyses were performed on an Agilent 6520 ESI-QTOF mass spectrometer with an Agilent Zorbax column (300SB C3, 2.1 x 150 mm, 5 µm). Mobile phase A was 0.1% formic acid in water and mobile phase B was 0.1% formic acid in LC-MS grade acetonitrile. The LC-MS method was as follows: 1% B from 0 to 2 min, linear ramp from 1% B to 61% B from 2 to 11 min, 61% B to 99% B from 11 to 12 min and finally 3 min of post-time at 1% B for equilibration, flow rate: 0.8 mL/min. Some LC-MS analyses were performed on Agilent 6550 ESI-QTOF mass spectrometer with an Agilent Zorbax column (300SB-C3 2.1 x 150mm, 5 µm). Mobile phases were same as previous and the gradient was as follows: 1% B from 0 to 2min with a linear gradient from 1 to 95% B over 10 minutes, then 95% B for 1 minute with the MS on from 4 to 12 minutes and a flow rate of 0.5 mL/min. All Agilent system data were processed using the Agilent MassHunter software package. Y-axis in all chromatograms represents total ion current (TIC) unless noted.

LC-MS/MS analysis for the serum protein pulldown digest was performed using an EASY-nLC 1200 nano-LC system with an Orbitrap Fusion Lumos Tribrid Mass Spectrometer (both Thermo Fisher Scientific) as described, using a PepMap RSLC

C18 column (2 μm particle size, 15 cm × 50 μm ID; Thermo Fisher Scientific). Mobile phase A was 0.1% formic acid in ultrapure water (v/v) and mobile phase B was 80% acetonitrile, 0.1% formic acid in ultrapure water (v/v). The nano-LC method was as follows: 1% B ramping in a linear manner to 41% B in A over 120 minutes, flow rate: 300 nL/min. Data were processed using PEAKS proteomics software.

Serum stability LC-MS assay. Peptides were incubated at 5 µM in PBS with 5% mouse serum (Gibco) at 37 °C for 24 hours. At the indicated time points, a 5 µL aliquot of the reaction mixture was removed, transferred to a fresh 1.5 mL microcentrifuge tube, and lyophilized. These aliquots were then re-suspended in water with 0.1% TFA and analyzed via LC-MS using an Agilent 6550 ESI-QTOF and the method described above.

Proteolysis LC-MS Assay. Peptides were incubated at 5 µM in PBS with 0.5 ng proteinase K at 37 °C for 2 hours. At each time point, a 0.5 µL aliquot was removed, transferred to an LC-MS vial and flash frozen. They were then resuspended 50:50 water:acetonitrile with 0.1% trifluoroacetic acid and reaction analyzed on an Agilent 6550 iFunnel Q-TOF MS. Time points were taken at t = 0 min, 30 min, 60 min, and 120 min.

SI References

1. A. J. Mijalis, *et al.*, A fully automated flow-based approach for accelerated peptide synthesis. *Nat. Chem. Biol.* **13**, 464–466 (2017).

Table S1. List of antigen sequences

	Long antigen	Minimal epitope
gp100	AVGALEGPRNQDWLGVPRQL	EGPRNQDWL
Adpgk	PVHLELASMTNMELMSSIVHQQV	ASMTNMELM
E6	KQQLLRREVYDFAFRDL	VYDFAFRDL
E7	IDGPAGQAEPDRAHYNIVTF	RAHYNIVTF
Trp1	TEMFVTAPDNLGYMYEVQWP	TAPDNLGYM
Tbrg4	ARCAKSFAFLNWLNLPLFEAF	FAFLNWLNL
Nsl1	VIQLQRINLEVFSSLYRKADF	INLEVFSSL
Hspa14	LGEAAGAAGFNVLLLIHEPSA	AAGFNVLLL
M47	GRGHLLGRLAAIVGKQVLLGRKVVVVR	
M48	SHCHWNDLAVIPAGVVHNWDFEPRKVS	

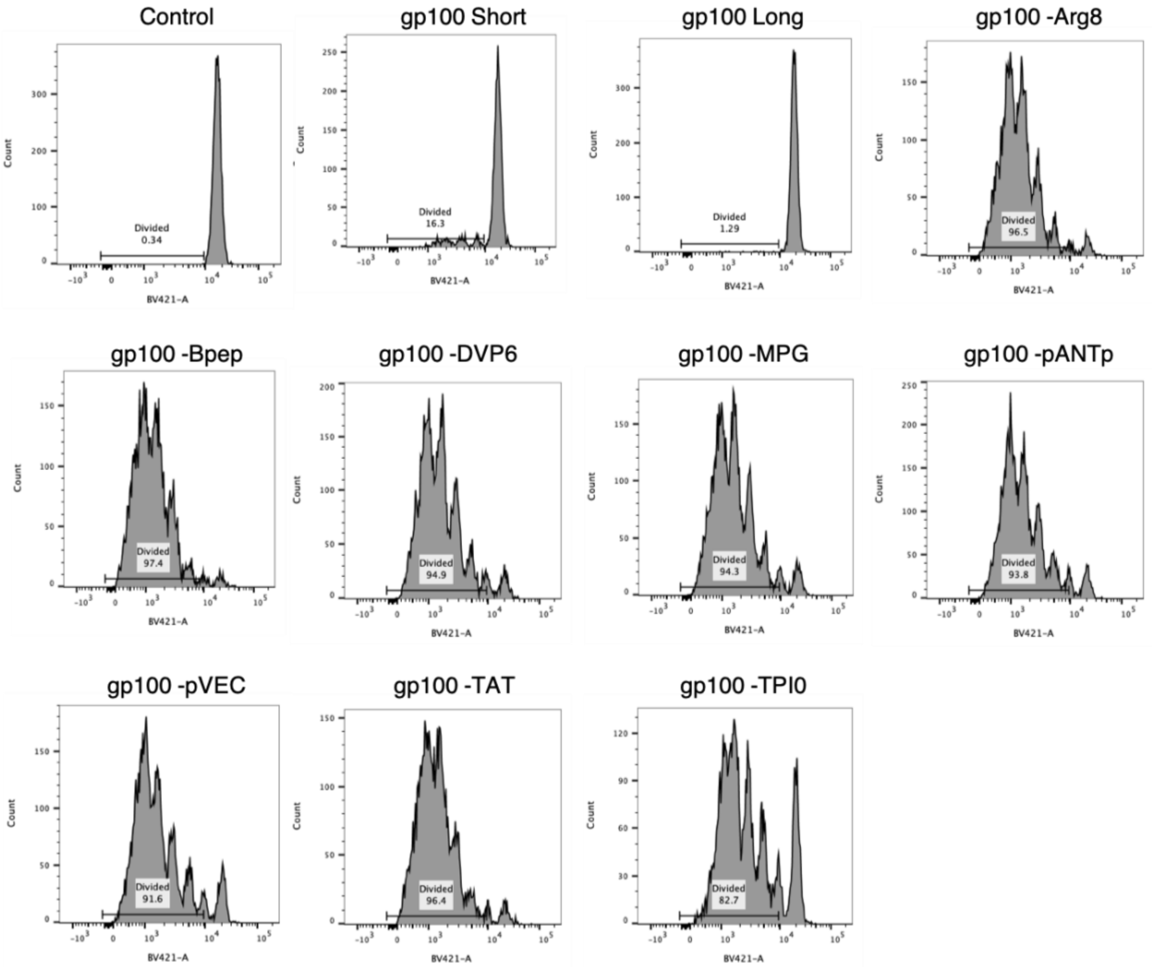

Figure S1. Representative cell trace violet proliferation histograms of pmel T cells after incubation with peptide loaded splenocytes. DCs were pulsed with peptide for 1 hour, before incubation with cell trace violet-labeled pmel T cells for 48 hours. Live cells were assessed for fluorescence and percentage division from the unstimulated control.

A.

CPP	Sequence
penetratin	RQIKIWFQNRRJKWKK
penetratin scramble	IKRQIKWKQRNWKFRJ
MPG	GLAFLGFLGAAGSTMGAWSQPKKKRKV
MPG scramble	FMKGALFGGSKTAQRLPKVSWGKAGLA

B.

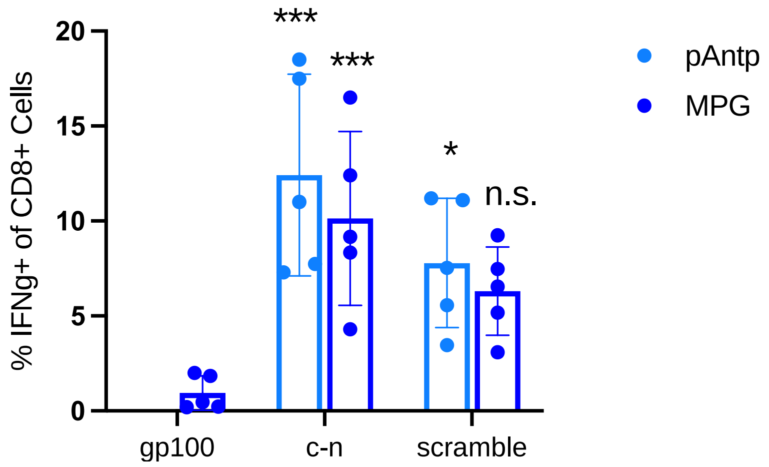

Figure S2. Comparing scrambled and original CPP sequences. A) Standard sequences of pAntp and MPG (used throughout this manuscript) and sequences of their scrambled variants. A random scrambler tool was used to generate these sequences and they were inspected to ensure that biophysical patterns present in the original sequences were not present in the scrambled variant. **B)** Mice (n=5) were immunized with 25 µg CDN and 5 nmol of either the long antigen (gp100), the indicated antigen-CPP with a click linkage (c-n), or the antigen click-conjugated to a randomly scrambled version of the indicated CPP on day 0 and day 14. The percentage of IFN-γ^+ T cells was determined via ICS on Day 21. A one-way ANOVA was performed comparing each construct to gp100. *** $p < 0.001$, * $p < 0.05$, n.s. not significant. In addition, a *t*-test comparing the c-n and scrambled versions of each CPP indicated they were not statistically different ($p > 0.05$).

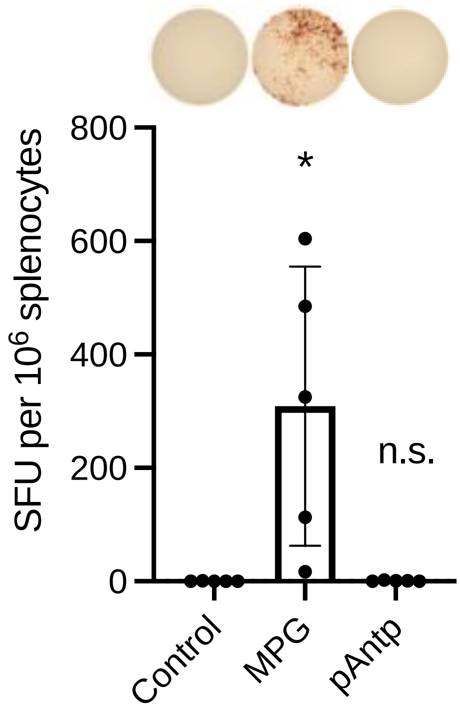

Figure S3. **A T cell response is elicited in C57Bl/6 mice against the MPG CPP sequence but not pAntp.** Mice (n=5) were immunized on day 0 and day 14 with gp100-MPG or gp100-pAntp. Spleens were resected and ELISPOT was performed on splenocytes harvested on day 21 using MPG or pAntp (no gp100 epitope) to stimulate splenocytes. The control data represents samples from unvaccinated mice stimulated with both MPG and pAntp.

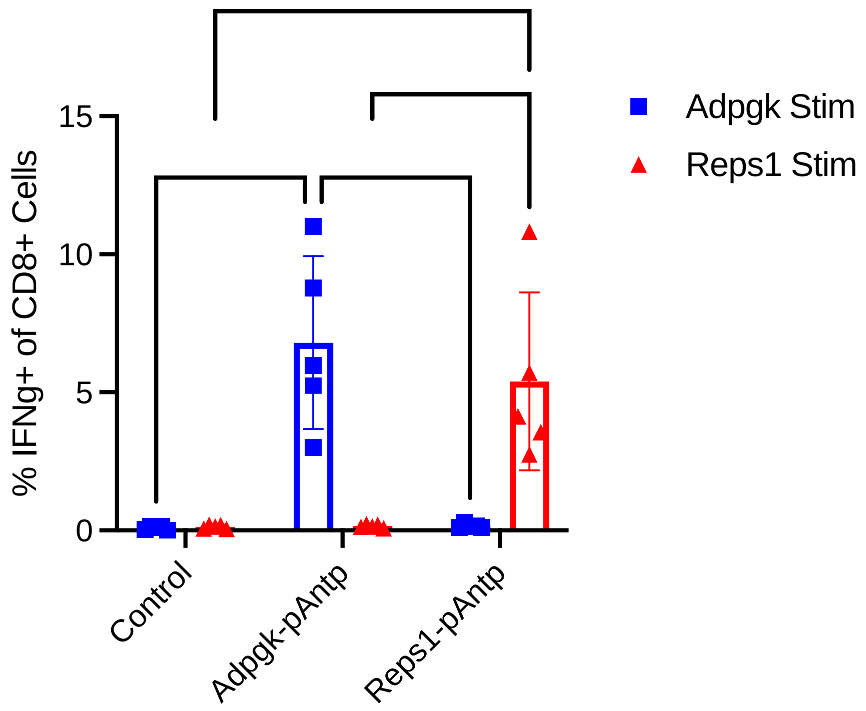

Figure S4. T cell response after stimulation with the optimal peptide epitope as determined by intracellular cytokine staining. Peripheral blood from 5 animals that had undergone prime and boost with either Adpgk-pAntp or Reps1-pAntp was stimulated with the matched cognate optimal epitope or a mismatched epitope, and then underwent intracellular staining to detect IFN-γ production by flow cytometry. Shown are mean ± standard deviation ***, $p < 0.0002$; ****, $p < 0.0001$ by 2way ANOVA followed by a Tukey post hoc test.

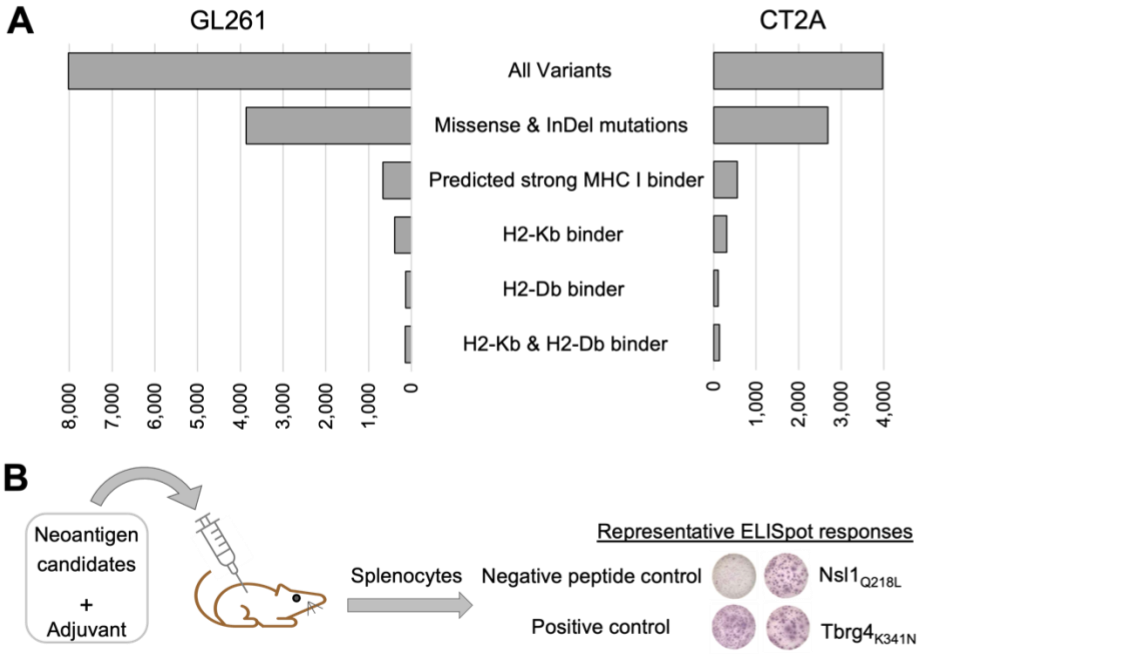

C

Tumor line	Neoantigen	DNA VAF	RNA VAF	TPM	H2 allele	Binding affinity percentile	Peptide sequence
GL261	$Nsl1_{Q218L}$	72%	64%	10.3	Kb	0.07	VIQLQRIN**L**EVFSSLYRKADFRQIKIWFQNRRMKWKK
CT2A	$Tbrg4_{K341N}$	47%	47%	8.8	Kb & Db	0.03	AKSFAFL**N**WLNLPLFEAFTQHRQIKIWFQNRRMKWKK
GL261	$Hspa14_{R168L}$	69%	55%	59.9	Kb & Db	0.16	LGEAAGAAGFNVL**L**LIHEPSARQIKIWFQNRRMKWKK
CT2A	$Hspa14_{R168L}$	38%	38%	44.7	Kb & Db	0.16	LGEAAGAAGFNVL**L**LIHEPSARQIKIWFQNRRMKWKK

Minimal epitope

SLP (21mer)

CPP (37mer)

Figure S5. Identification of neoantigens from GL261 and CT2A. **A)** Cancer genome analysis of whole exome sequencing (GENEWIZ, Inc.; Illumina HiSeq 2x150bp) of *in vitro* GL261 and CT-2A syngeneic murine cancer lines (n=2 each), using standard pre-processing (Picard tools), alignment to the mm10 reference genome (bwa tool), and C57BL/6 tissue as the germline comparison. Displayed are the number of total variants (called by a consensus of mutect and strelka tools, and shared by both replicates), missense and InDel variants (identified by VEP annotations), and predicted strong MHC I binders (defined as ic50 binding affinity<1,000 and binding affinity percentile<0.5; using consensus predictions from NetMHCpan, NetMHC, and PickPocket tools) – stratified by H2 alleles. Neoantigen candidates were prioritized from among predicted strong binders based on the variant allele frequency (VAF) in both DNA and RNA sequencing, gene expression (transcripts per million; TPM), and synthesizability of the neoantigen peptide. **B)** For screening of candidate neoantigens' immunogenicity, C57BL/6 mice were immunized subcutaneously with 100 µg of select neoantigen minimal epitopes (dissolved in DMSO) and 100 µg of Complete Freund's Adjuvant. Spleens were collected on day 10 and processed for IFNγ ELISpot (Mabtech, AB.). Representative responses are displayed (200,000 splenocytes per well, stimulated with cognate minimal epitopes for 36-48 hours), and compared to negative (SIINFEKL peptide) and positive (phytohemagglutinin) controls. **C)** Three confirmed neoantigens (all missense variants) were selected for further experimentation – including one from GL261, one from CT-2A, and one present in both lines. For each, 21-mer synthetic long peptide (SLP) and 37-mer cell penetrating peptide (CPP) versions were synthesized (GenScript, Co.; with all SLP and CPP purities confirmed by HPLC to be ≥90%). The neoantigen's amino acid substitution is indicated by bold underline. The 21-mer SLP includes the minimal epitope sequence (red) and flanking native peptide sequence (black). The CPP version consists of the SLP with a C-terminal RQIKIWFQNRRMKWKK sequence (blue). Average VAF and TPM values are reported from two replicates.

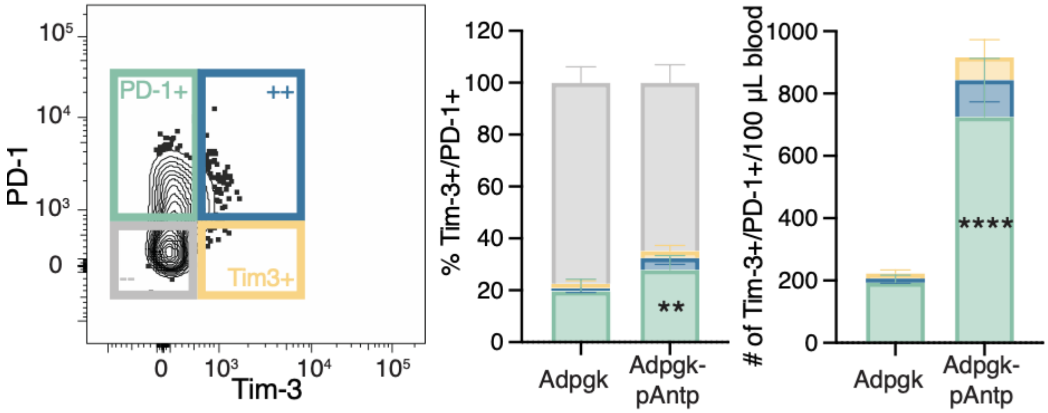

Figure S6. Exhaustion phenotyping on peripheral blood after a prime and boost in naïve mice. C57Bl/6 mice (n = 5 animals/group) were immunized with 5 nmol Adpgk or Adpgk-pAntp peptide combined with 25 µg cyclic-di-GMP on days 0 and 14, and then on day 21 peripheral blood was restimulated with optimal Adpgk peptide *ex vivo*, permeabilized for intracellular cytokine staining to identify antigen-specific IFN-γ^+ cells and stained for cell surface phenotypic markers. Representative flow cytometry plots, percentages, and quantification of PD-1/TIM3 expression. Statistical analyses were performed using a two-way ANOVA with Šídák's multiple comparisons test.

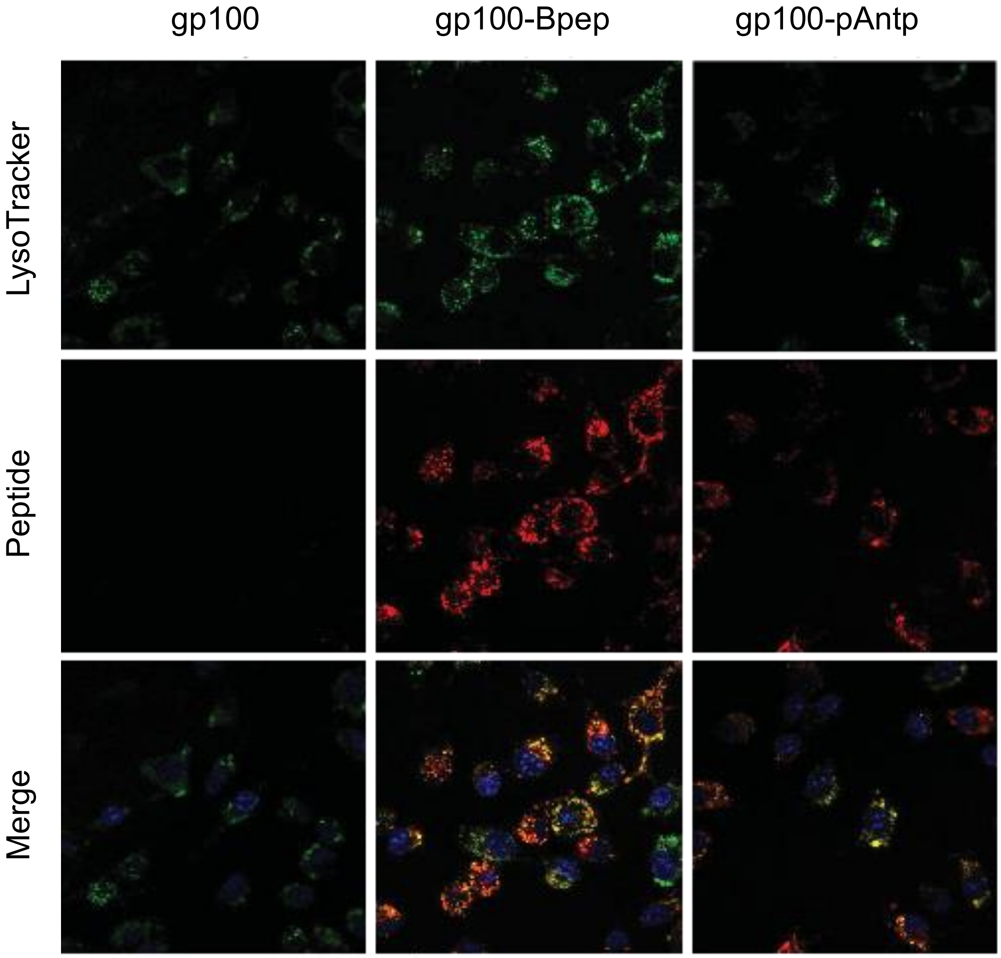

Figure S7. CPPs increase intracellular delivery. Confocal imaging of SulfoCy5-labeled EGP and EGP-CPP uptake by DC2.4 cells after 2.5 µM incubation for 1 hour. Cells were also stained with Hoescht and LysoTracker Green DND-26.

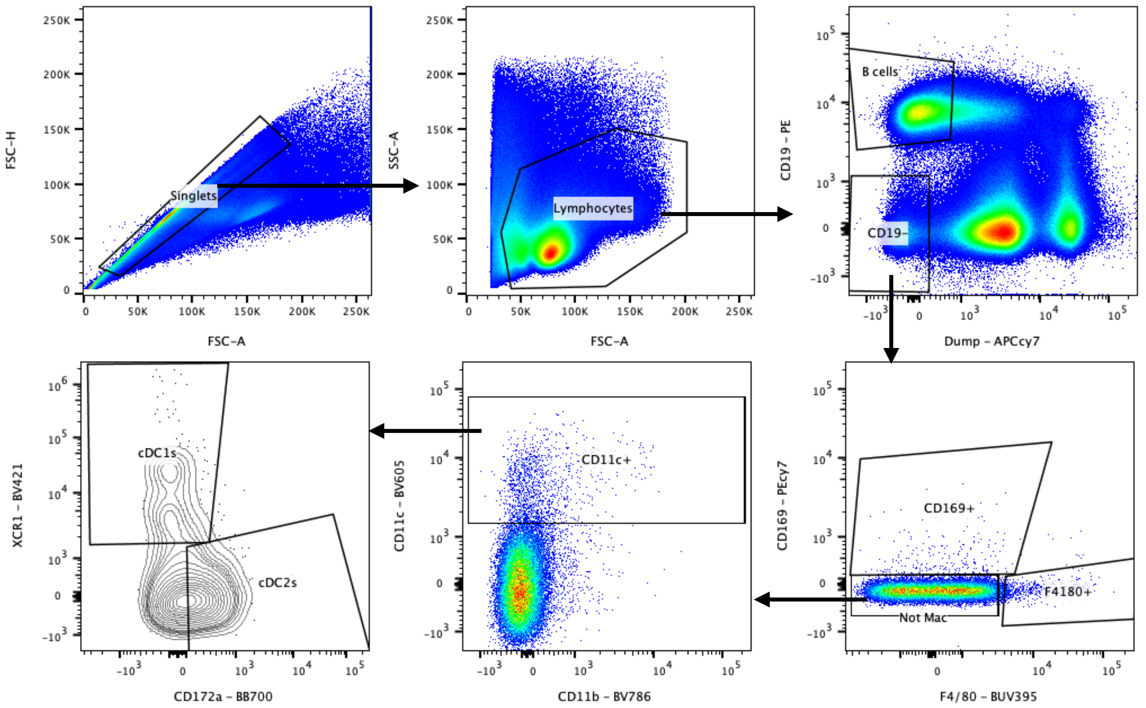

Figure S8. Gating strategy for subtyping antigen presenting cells in the inguinal lymph node. In brief, singlet events were first gated for negative expression of live/dead, NK1.1, CD3, and Ly6G. B lymphocytes were identified by expression of CD19, while CD19- cells were further subdivided. Macrophages were identified as F4/80+ or CD169+. Non-macrophage CD11c+ cells were further subdivided into XCR1+ cDC1s and CD172a+ cDC2s. Within these populations, antigen uptake was assessed via percentage of cells that had cy5 signal. Additionally, CD11c+ cells were assessed for expression levels of MHCII and CD86.

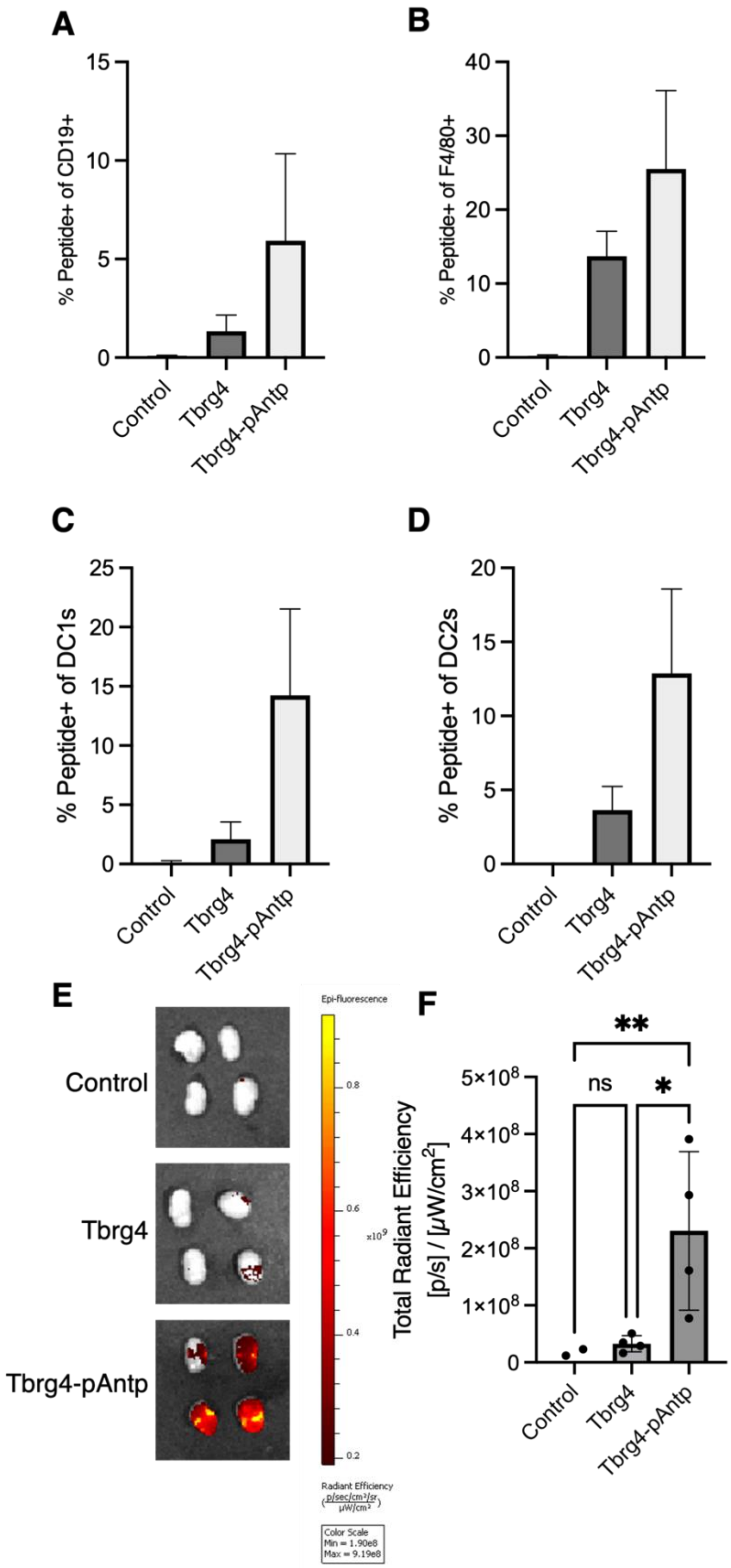

Figure S9. Tbrg4 conjugated to pAntp exhibits increased trafficking to draining lymph nodes and association with professional antigen presenting cells. Quantification of % peptide$^+$ cells at 48 hours post immunization as determined by flow cytometry for CD19+ B cells (A), F4/80+ macrophages (B), DC1s (C), and DC2s (D). (E) Whole-tissue fluorescence imaging of inguinal lymph nodes 48 hr after immunization with 25 nmol labeled Tbrg4 or Tbrg4-pAntp with 25 µg of cyclic-di-GMP (*n* = 4 LNs per group) and quantification of the total radiant efficiency (F).

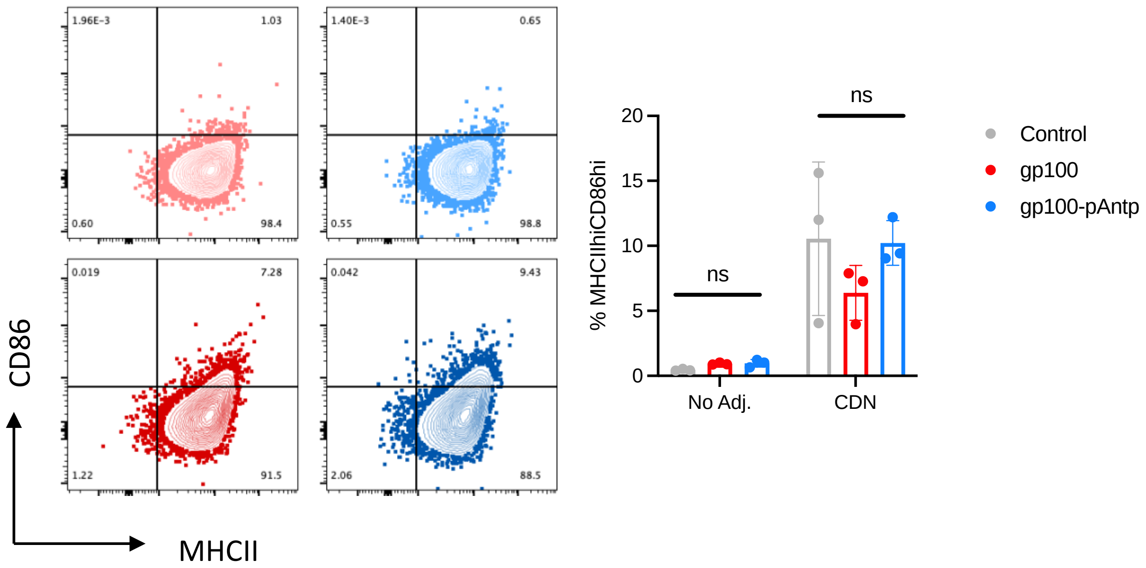

Figure S10. Upregulation of CD86 and MHCII on B cells is independent of peptide sequence. C57Bl/6 mice were injected with cy5-labeled gp100 or gp100-pAntp in the presence and absence of c-di-GMP (CDN). At 48 hours, B cells in the dLN did not upregulate CD86 and MHCII in response to the CPP compared with gp100 alone. The addition of adjuvant caused upregulation of both markers. The percentage of MHCIIhiCD86hi cells was statistically compared using a 2 way ANOVA with a Tukey post hoc test.

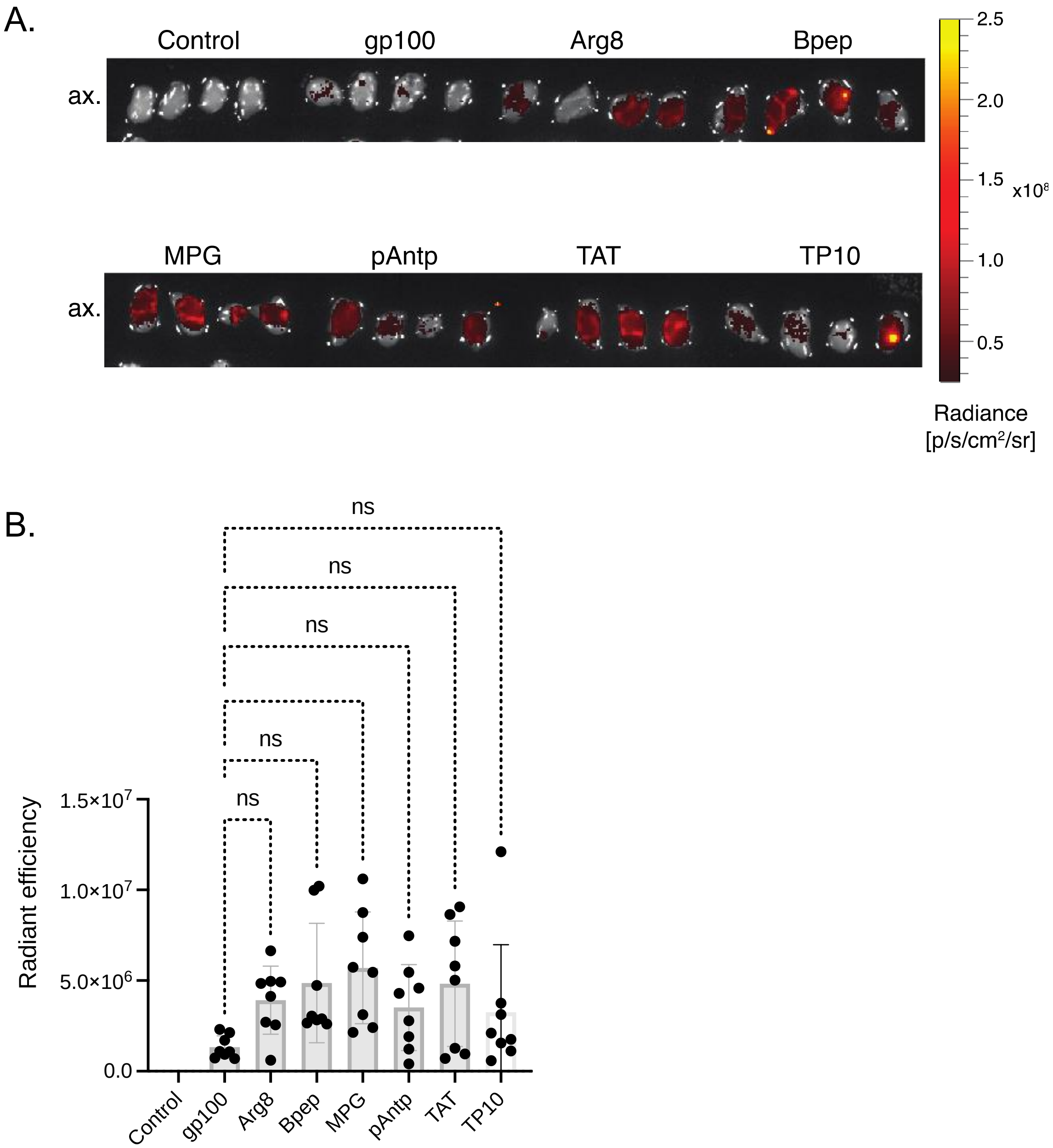

Figure S11. CPPs do not alter accumulation in the axillary lymph node. Mice (n=4) were immunized s.c. at the tail base with 25 nmol fluor-gp100 or fluor-gp100-CPP (denoted by the CPP name) and CDN. Axillary (shown) and inguinal lymph nodes were resected after 48h for whole tissue fluorescence imaging. A) Images of four axillary lymph nodes for each antigen construct. B) Plot comparing radiant intensities for all axillary lymph node samples. * denotes $p < 0.05$, n.s. not significant.

A.

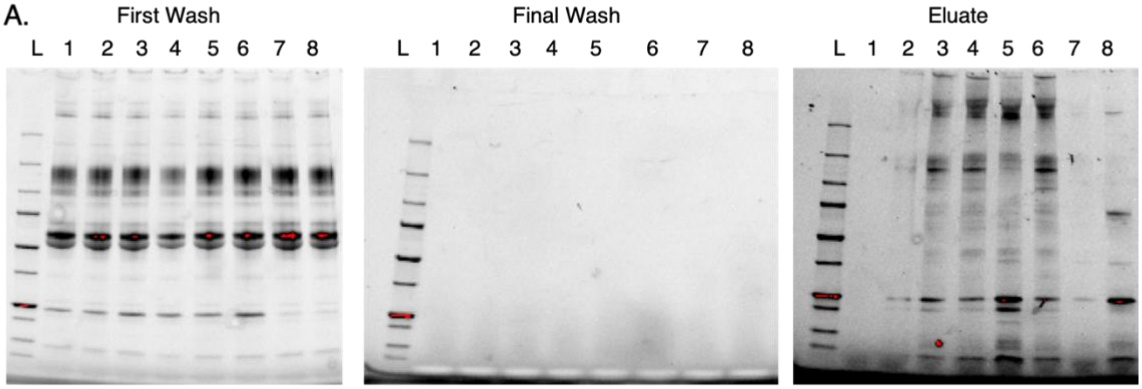

1. Serum control
2. Antigen
3. Arg_8
4. Bpep
5. pAntp
6. TAT
7. TP10
8. MPG (C-N)

B.

Band #	Protein	-10log(*p*)	% Coverage	Identified Peptides	Unique Peptides	MW identified protein (kDa)	MW assembled complex (kDa)
1	Immunoglobulin A	308.67	80	75	74	36.9	320
1	Apolipoprotein B-100	289.92	25	99	66	50.9	-
2	Carboxylesterase 1C	222.01	54	34	30	62.1	180
2	Hemoglobin subunit B-1	230.93	94	26	8	15.8	64.5
2	Hemoglobin subunit A	195.53	93	22	4	15.1	64.5
2	Hemoglobin subunit B-2	193.83	79	22	4	15.9	64.5
3	Apolipoprotein A1	311.59	71	46	38	30.6	-

Figure S12. Identification of serum proteins that bind antigen-CPPs. A) First wash, final wash, and elution native PAGE gels from experiment in figure 5D. Briefly, biotinylated CPPs were bound to streptavidin beads, then incubated in 50% mouse serum for 1 hour. The beads were then washed using PBS, analyzing each wash via native PAGE until no further serum proteins were observed in the eluate. Bound proteins were then eluted in mild acid and analyzed by native PAGE. **B)** Several prominent bands were excised from the elution gel in A, digested in trypsin, and analyzed via LC-MS/MS. PEAKS software was used to identify the proteins present in each band.

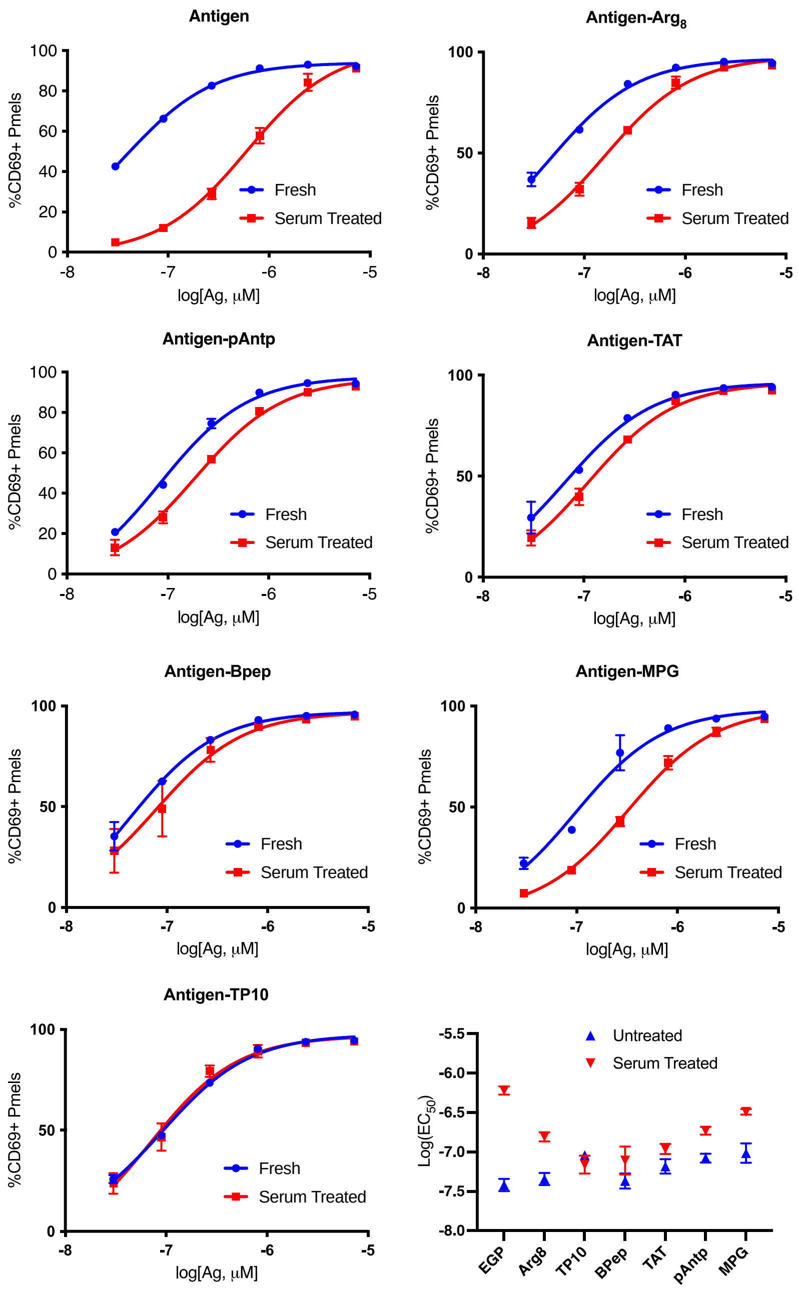

Figure S13. CPP conjugation confers protection in serum relative to the antigen alone. Extended serum stability data from Figure 6A-C**.** Each construct was either used fresh or incubated overnight in 10% serum, then serially diluted and used to stimulate pmel T cells in an *in vitro* activation assay. Shown are plots of pmel activation, measured via CD69 upregulation, against the concentration of the fresh or serum-treated construct. The difference between the fresh and serum treated plots indicates the extent of degradation in serum. This difference can also be visualized by plotting the log(EC_{50}) of the fresh and serum-treated samples for each construct.

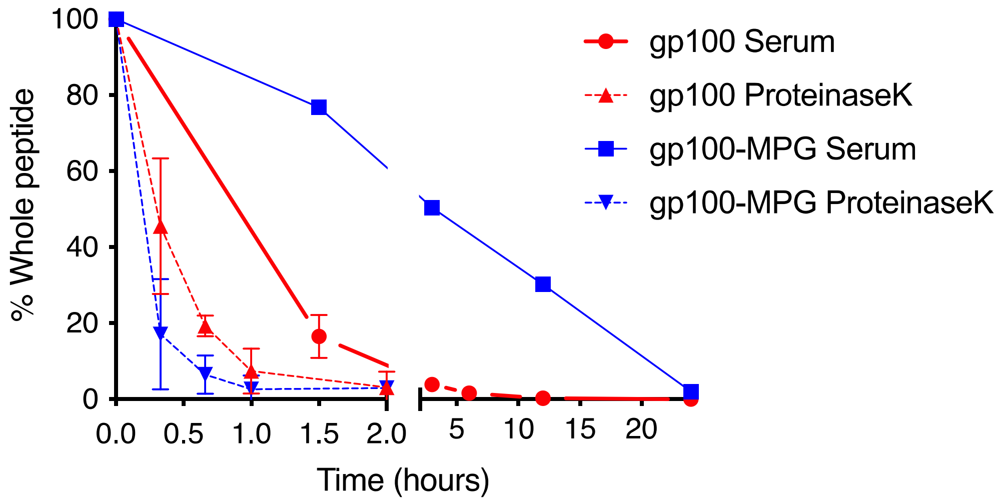

Figure S14. CPP conjugation does not confer protection against proteolysis by isolated proteinase K. Peptides (gp100 and gp100-MPG) were incubated at 5 µM in PBS with 0.5 ng proteinase K for 120 minutes. At 0, 20, 40, 60, and 120 min 5 µL aliquots were removed and analyzed via LC-MS to determine the relative amount of intact antigen construct remaining.

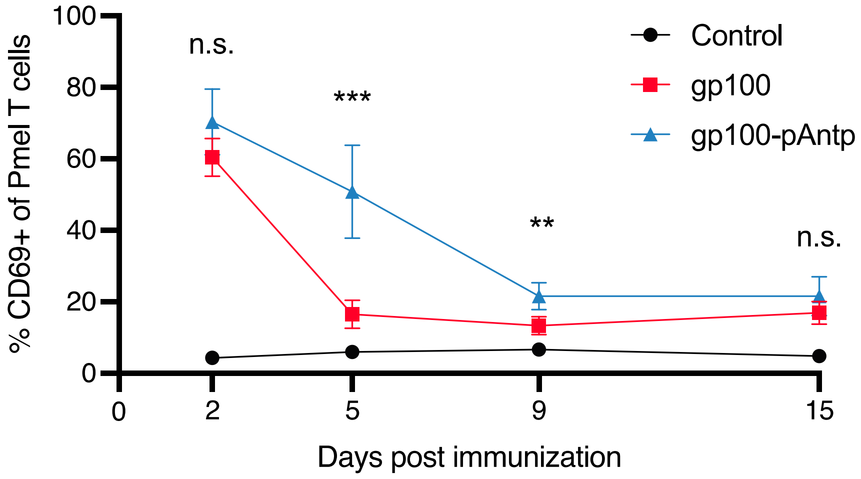

Figure S15. Mice were immunized with 25 µg CDN and 5 nmol of either EGP or EGP-pAntp on Days 0, 6, 10, or 13 (one group per time point). On day 14, 10^6 pmel-1 T cells were injected retro-orbitally. 24 hours later, axillary and inguinal lymph nodes were harvested and analyzed to determine CD69 upregulation. Data are shown for the axillary lymph nodes and the control line is derived from untreated spleens (inguinal lymph node data are given in Figure 6F).

Appendix. LC-MS characterization of peptides

gp100 long

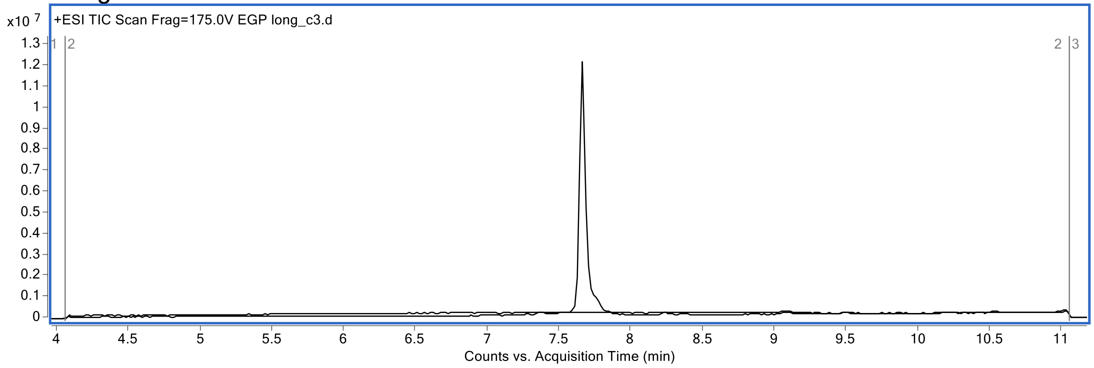

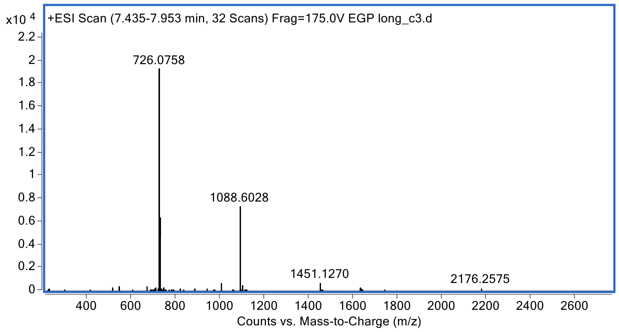

gp100-Arg8

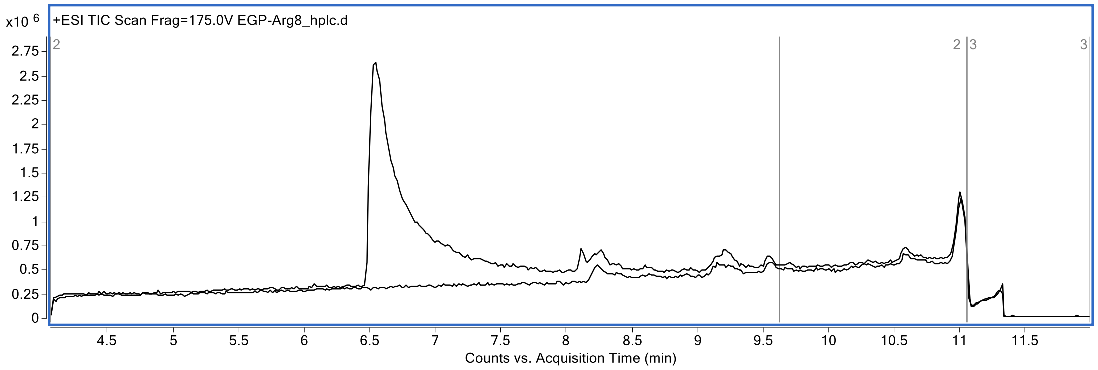

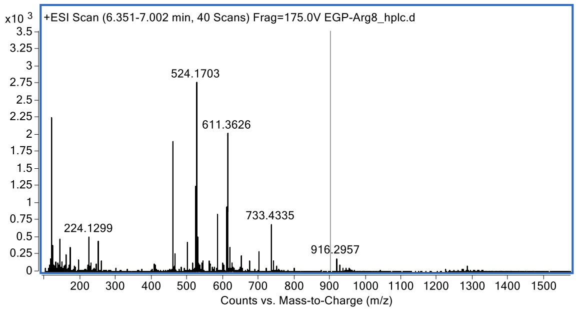

gp100-Bpep

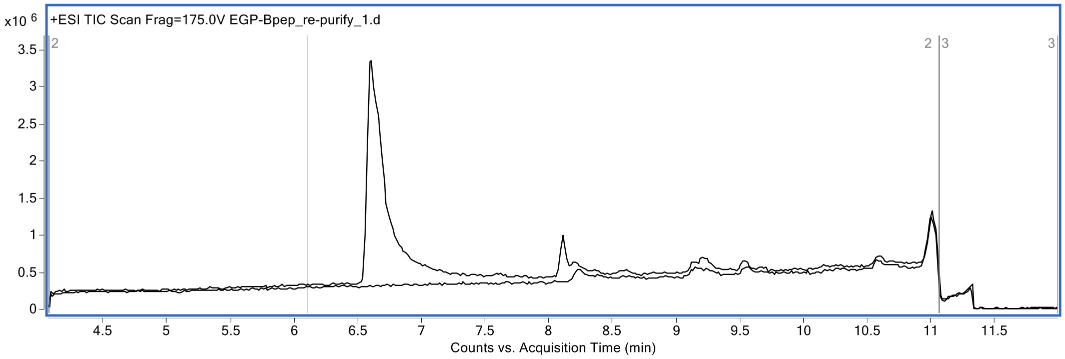

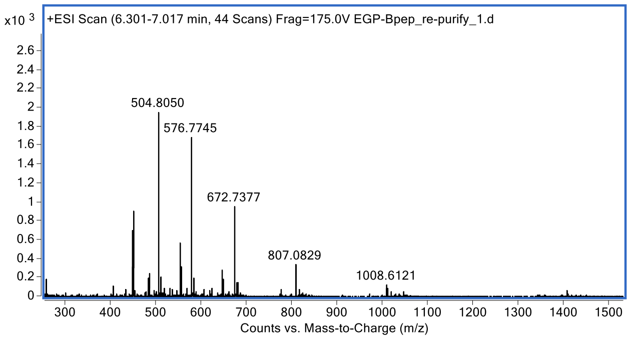

gp100-DPV6

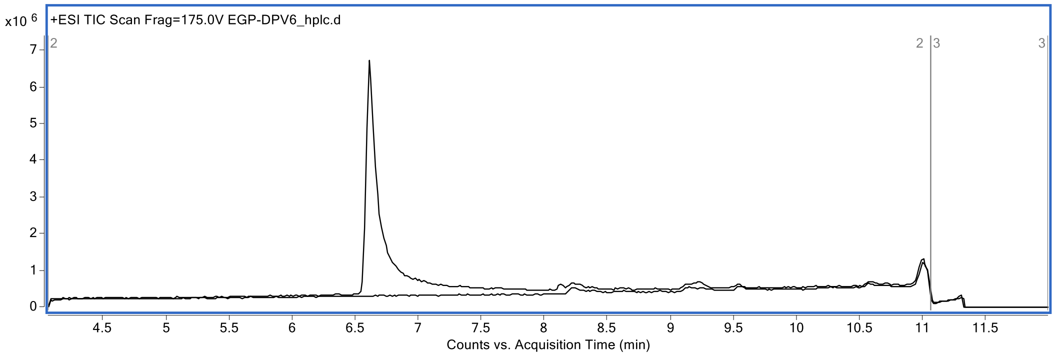

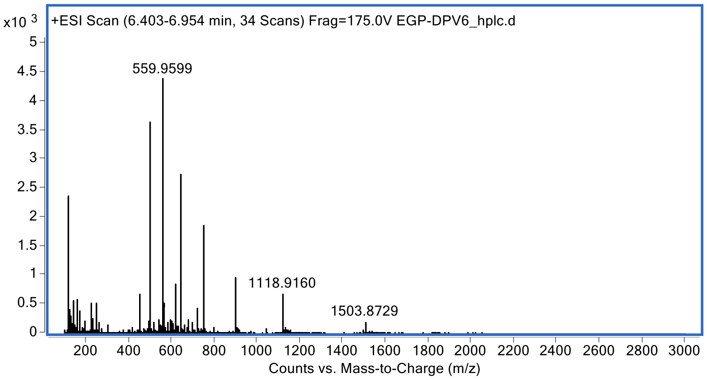

gp100-MPG single

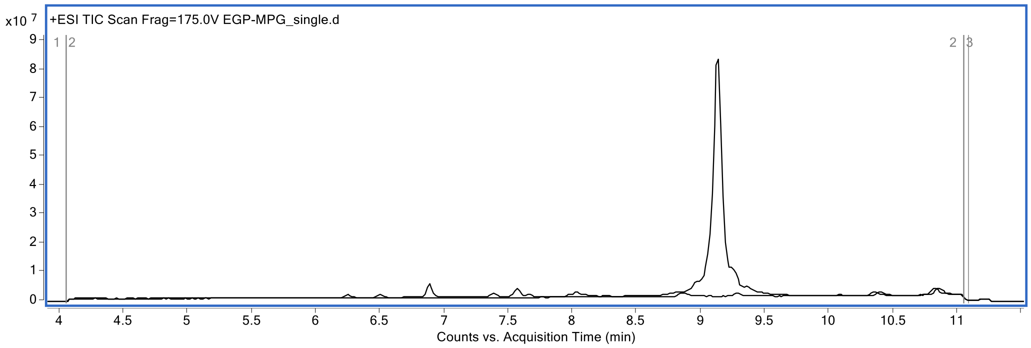

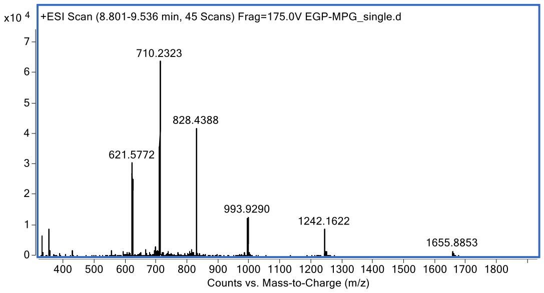

gp100-MPG C-N

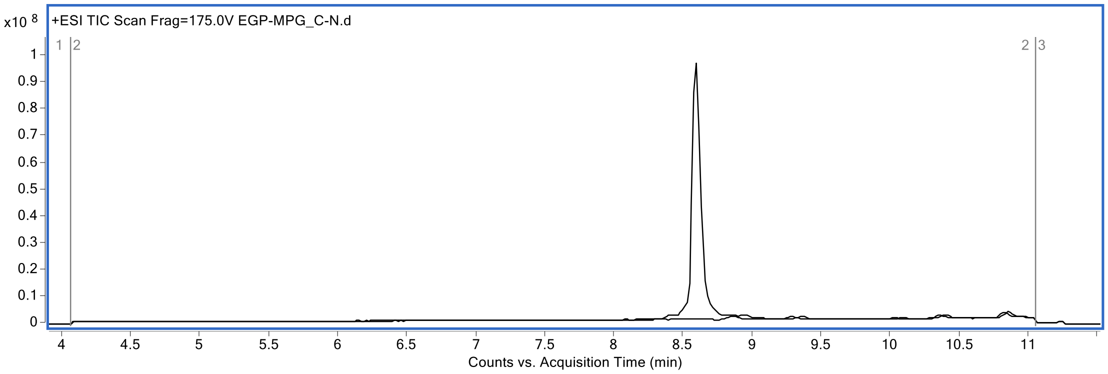

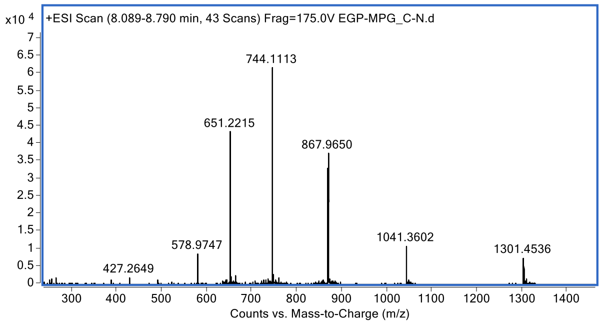

gp100-MPG C-C

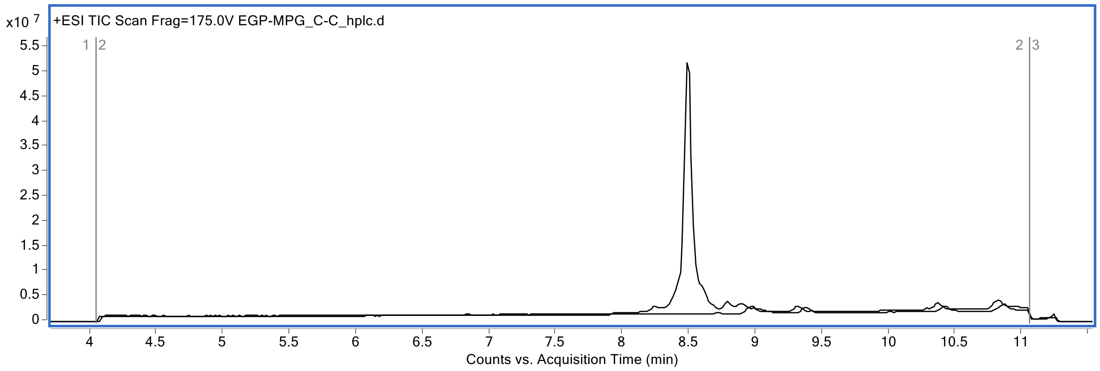

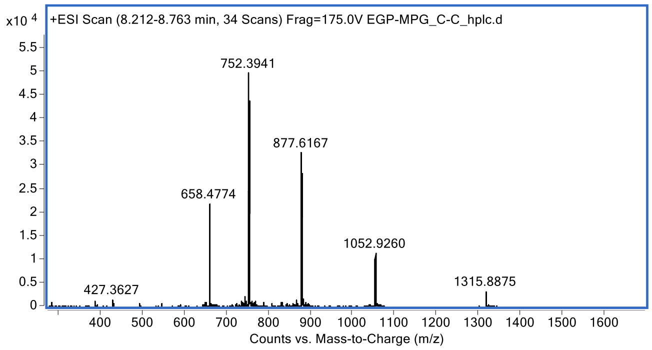

gp100-MPG scramble

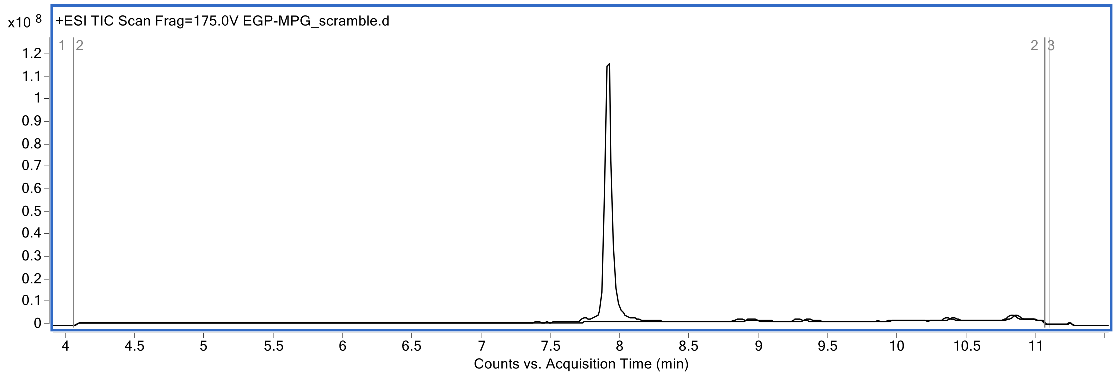

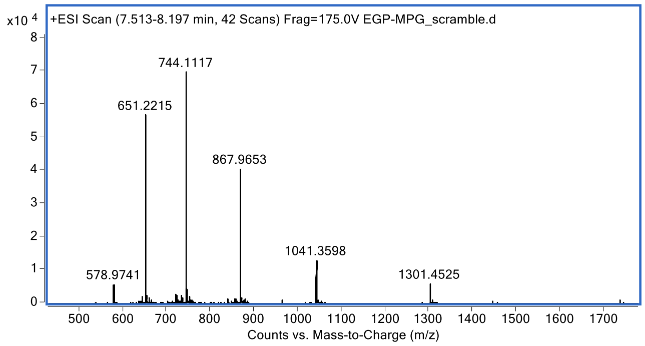

gp100-pAntp single

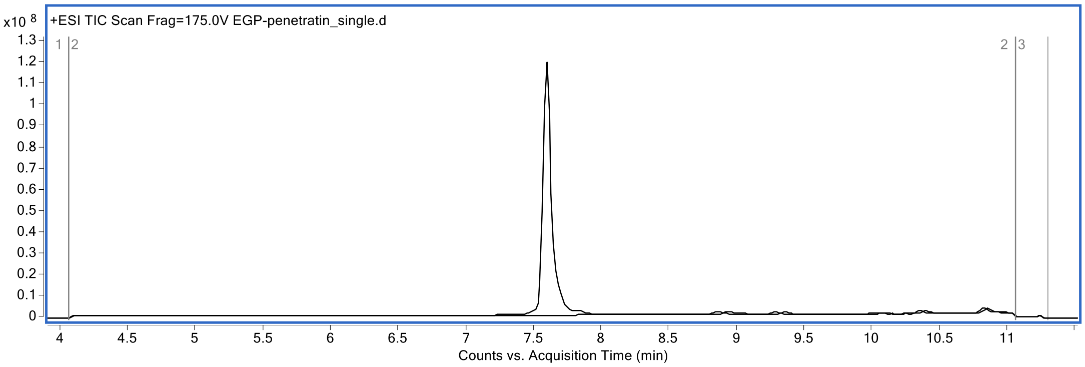

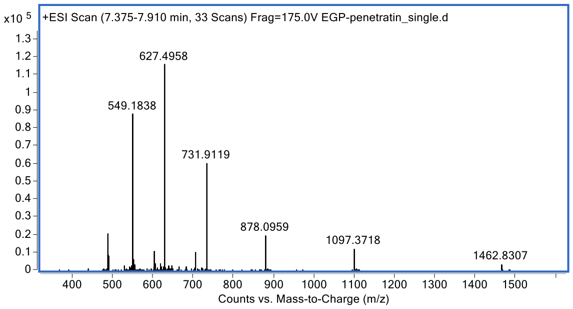

gp100-pAntp C-N

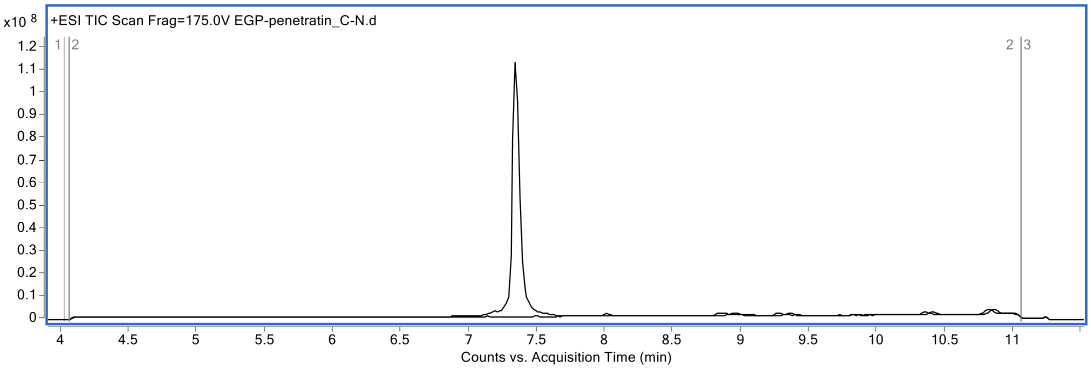

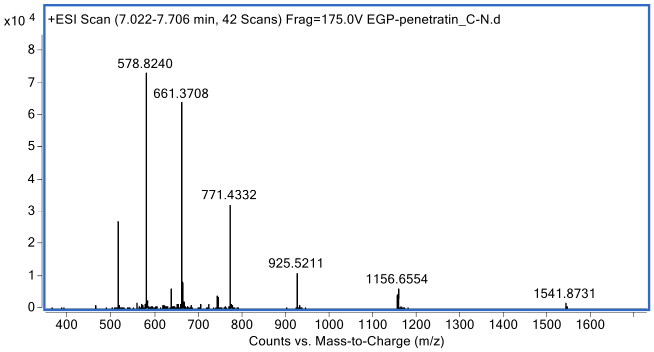

gp100-pAntp C-C

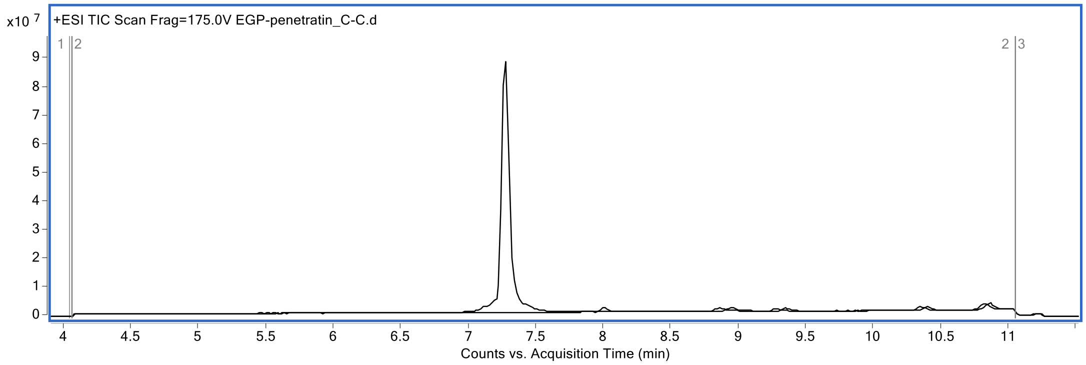

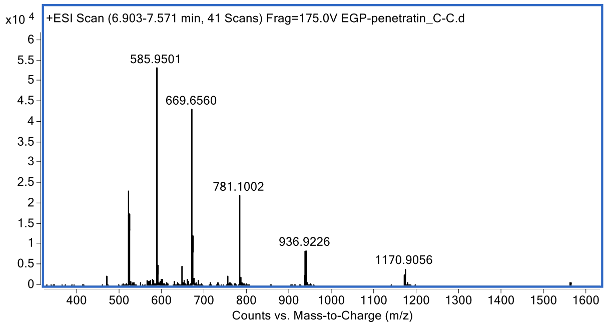

gp100-pAntp scramble

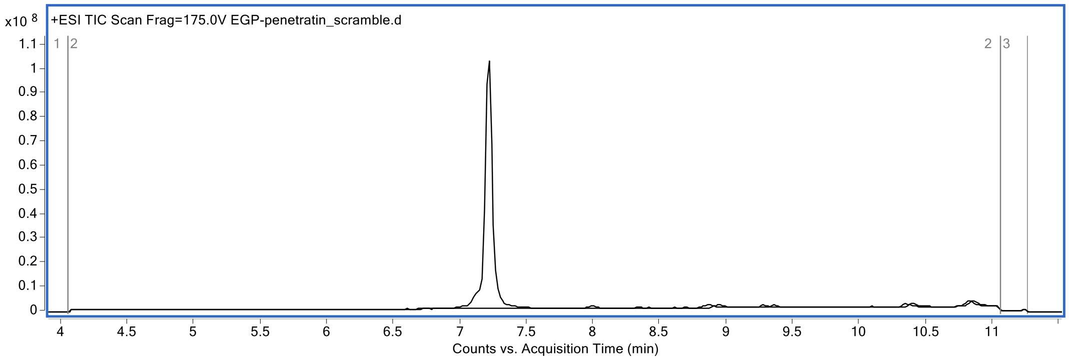

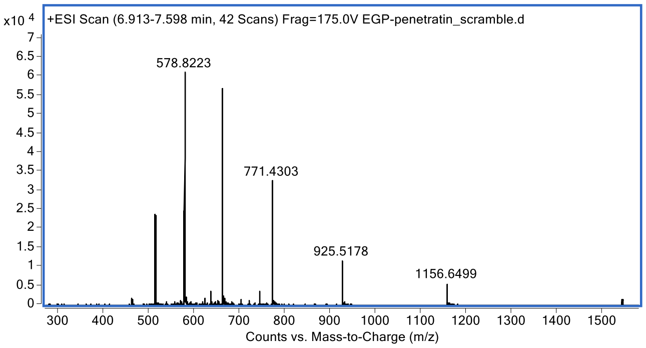

gp100-pVEC

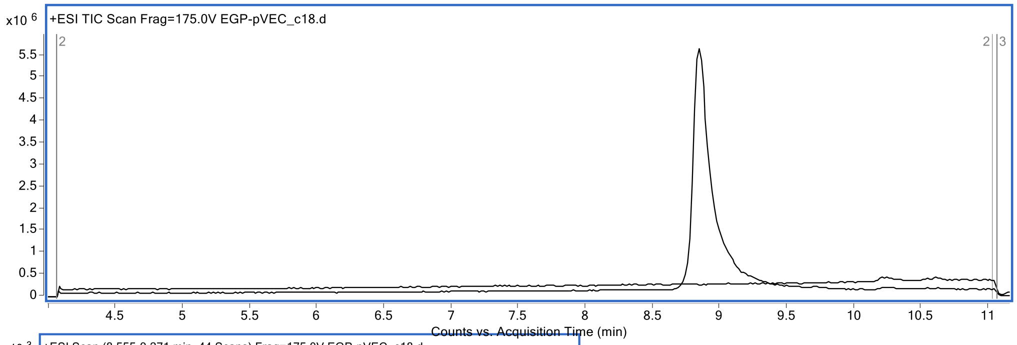

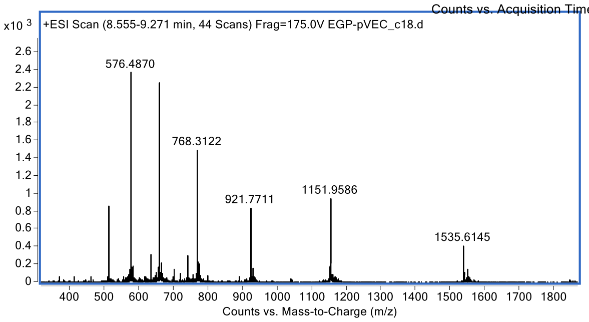

gp100-TAT

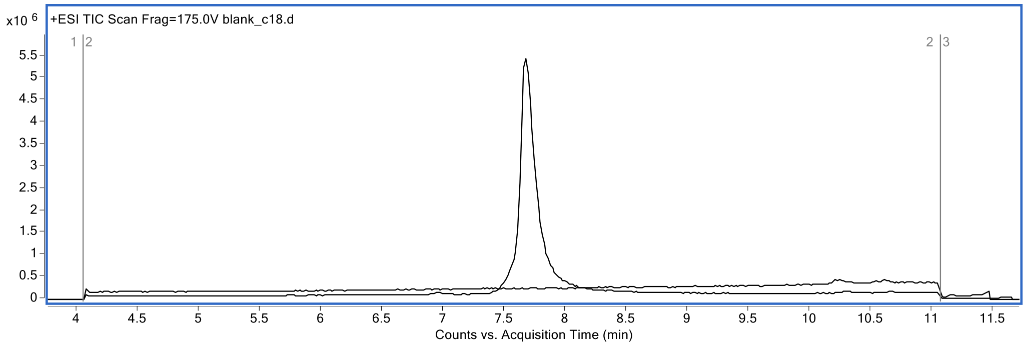

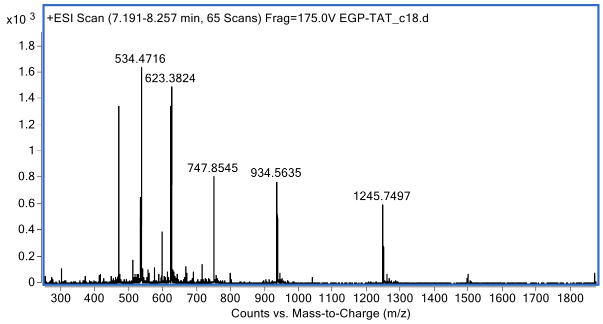

gp100-TP10

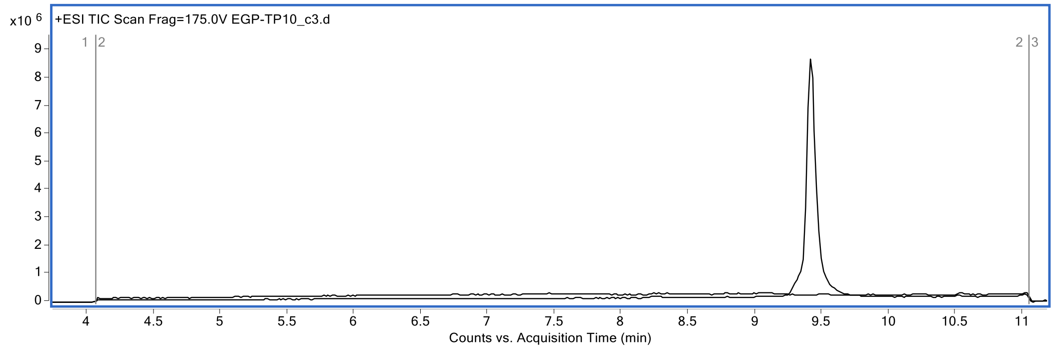

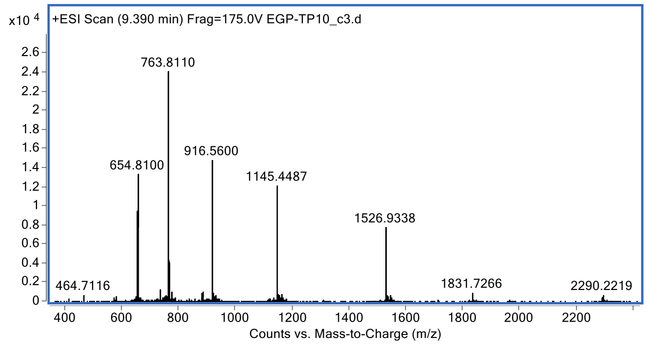

Adpgk long

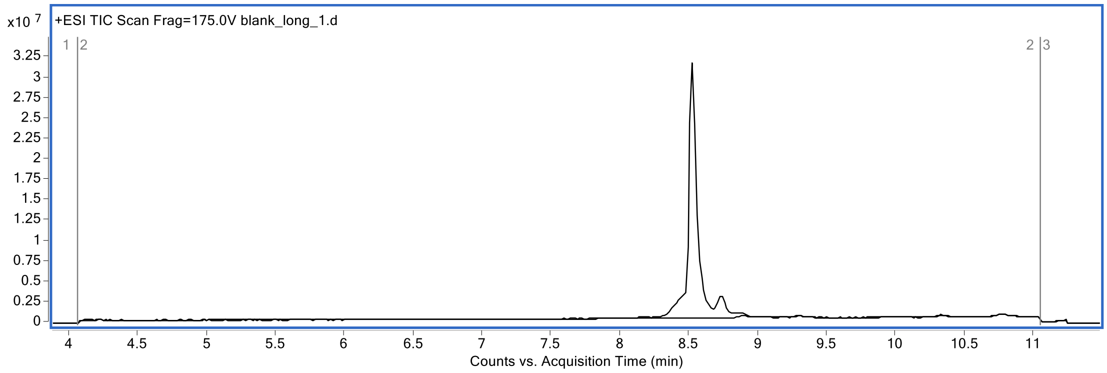

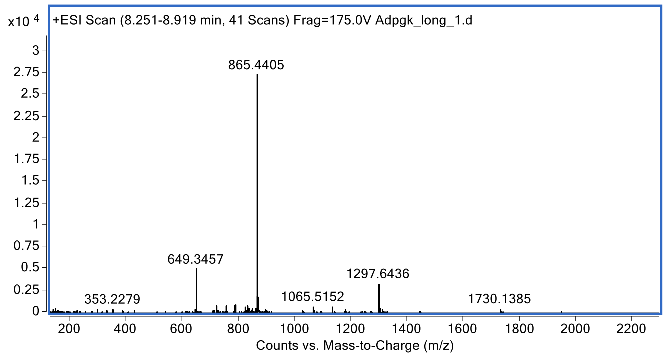

Adpgk-MPG

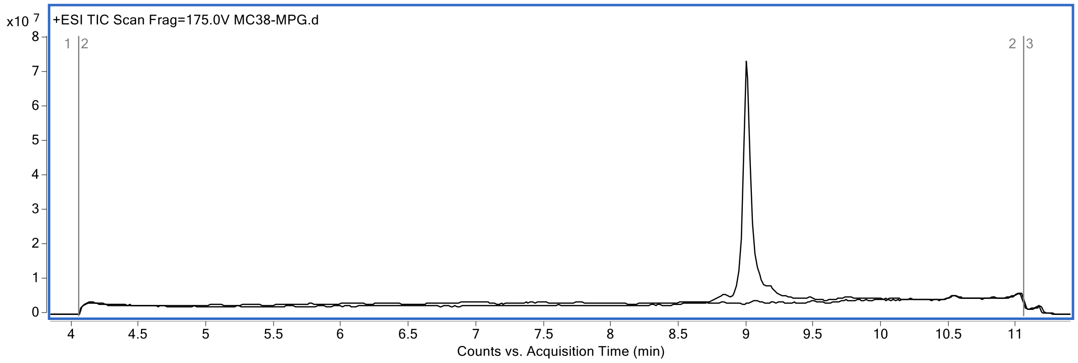

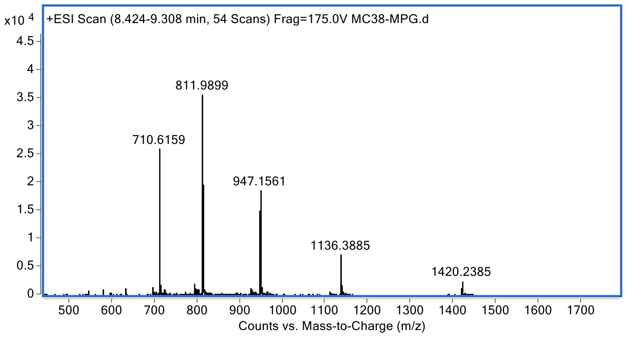

Adpgk-pAntp

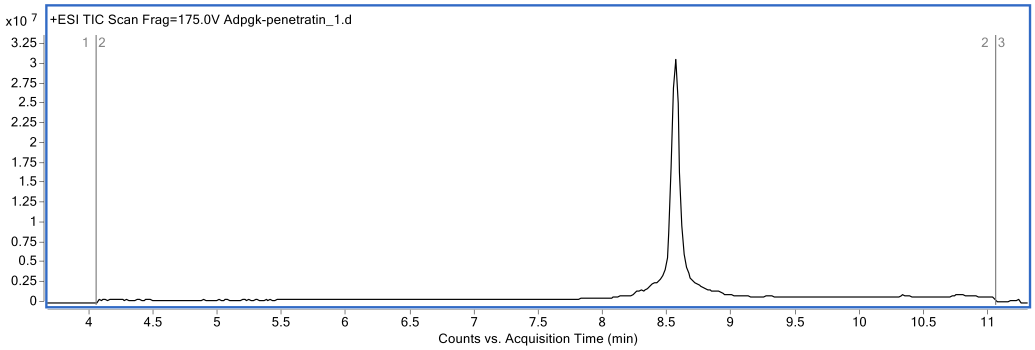

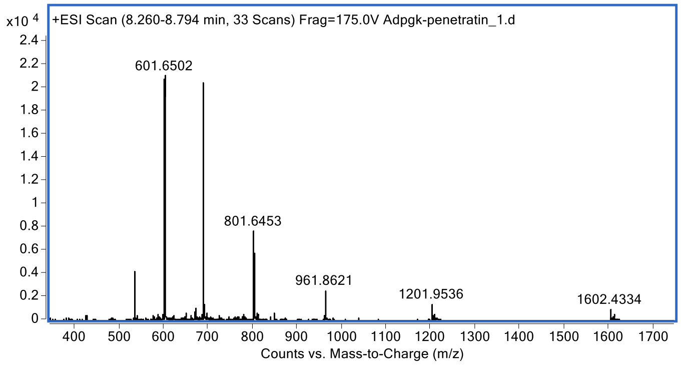

Adpgk-TAT

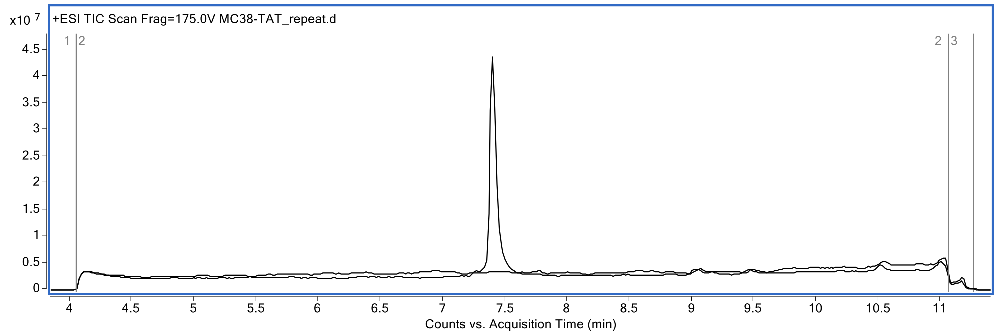

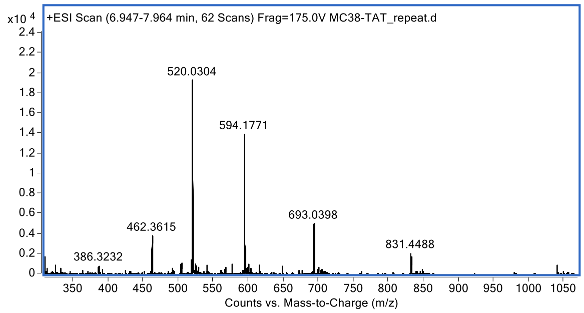

Adpgk-TP10

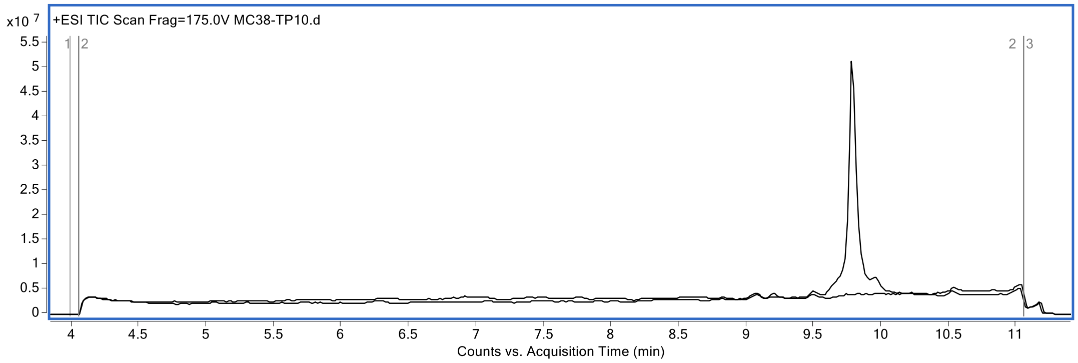

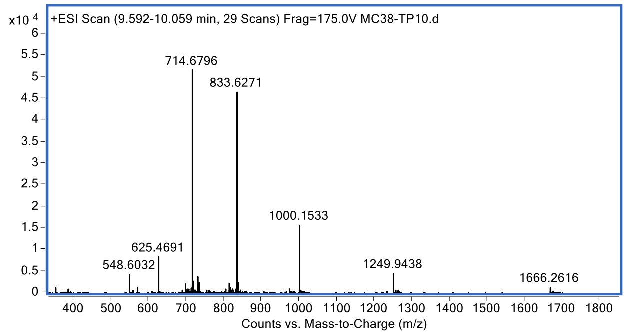

E6 long

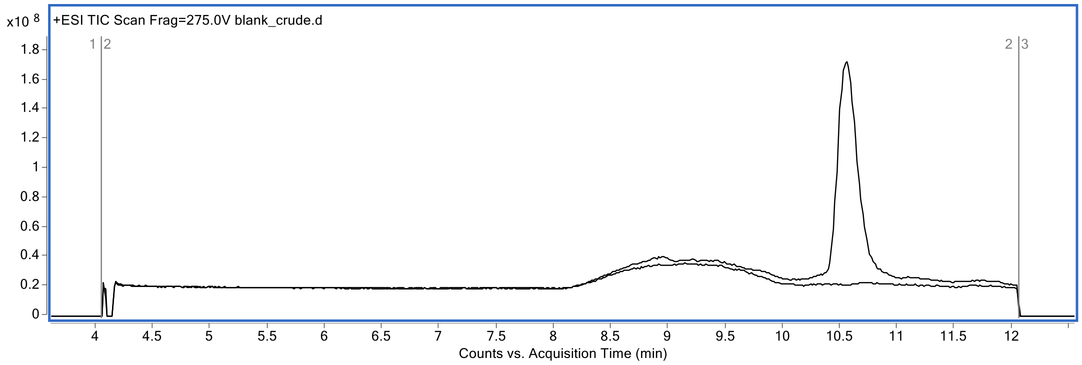

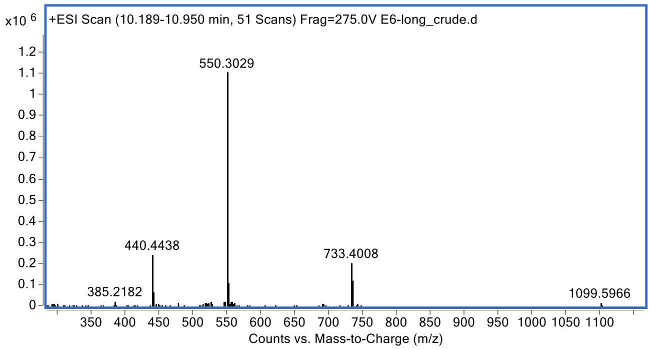

E6-pAntp

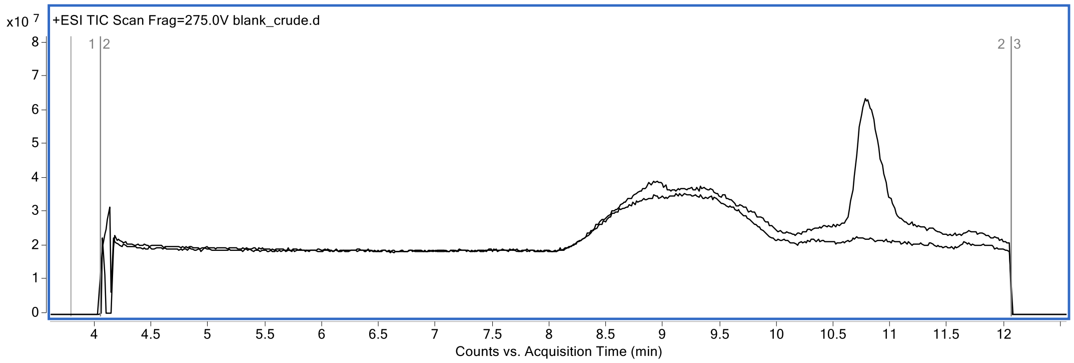

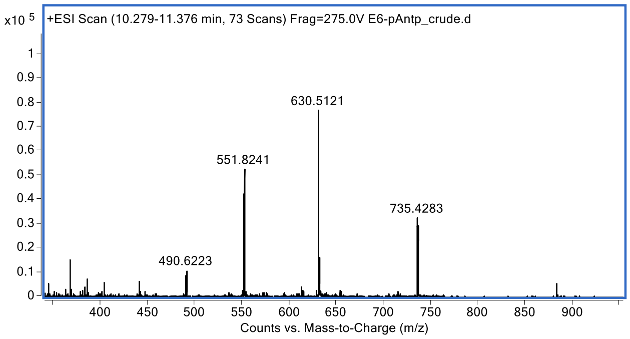

Trp1 long

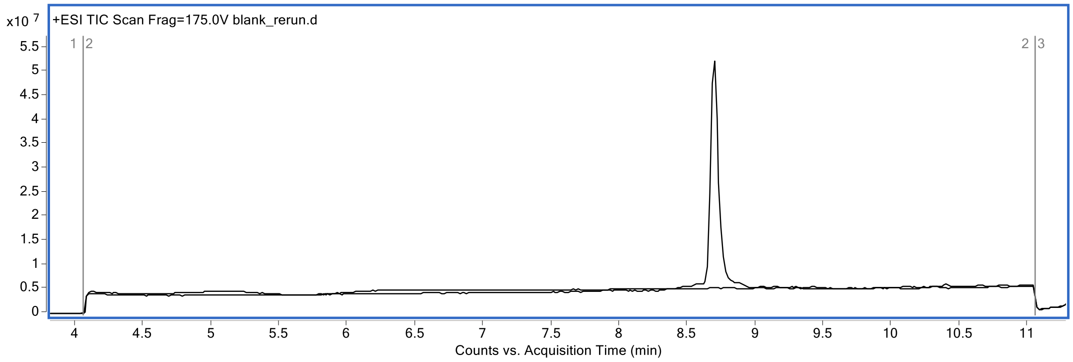

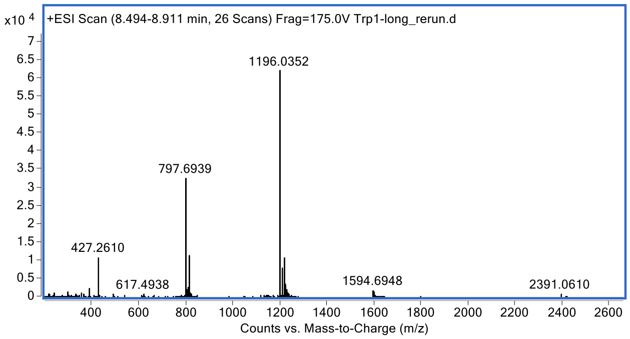

Trp1-pAntp

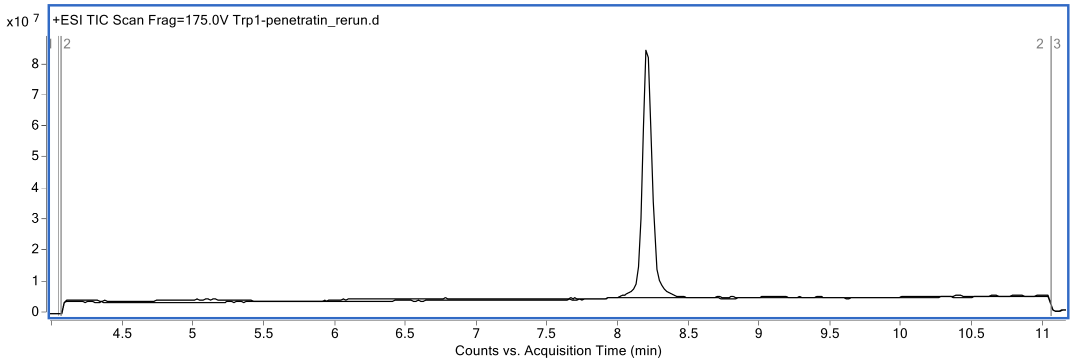

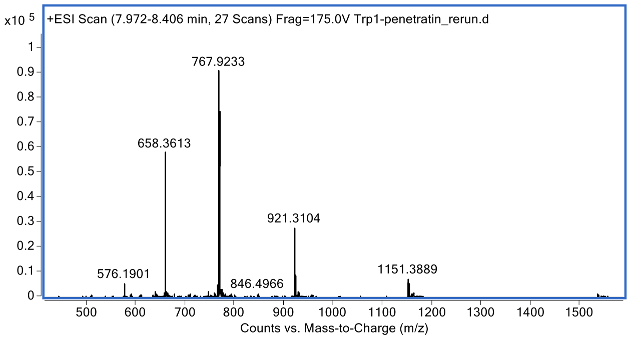

BDP-TR-gp100 long

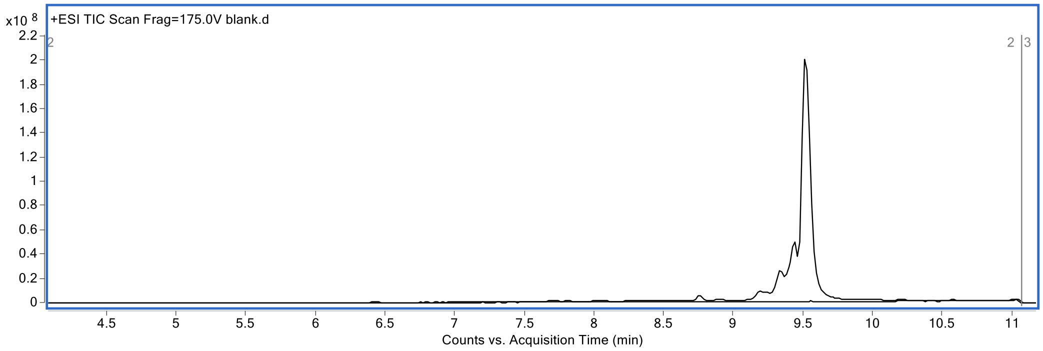

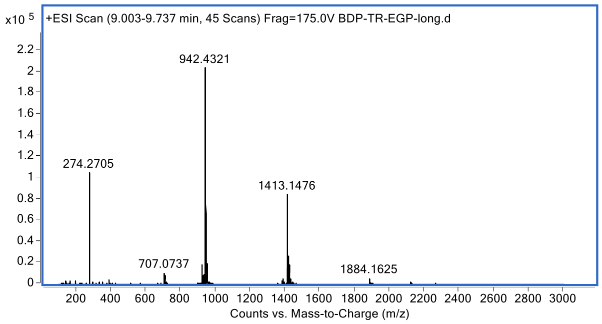

BDP-TR-gp100-Arg$_8$

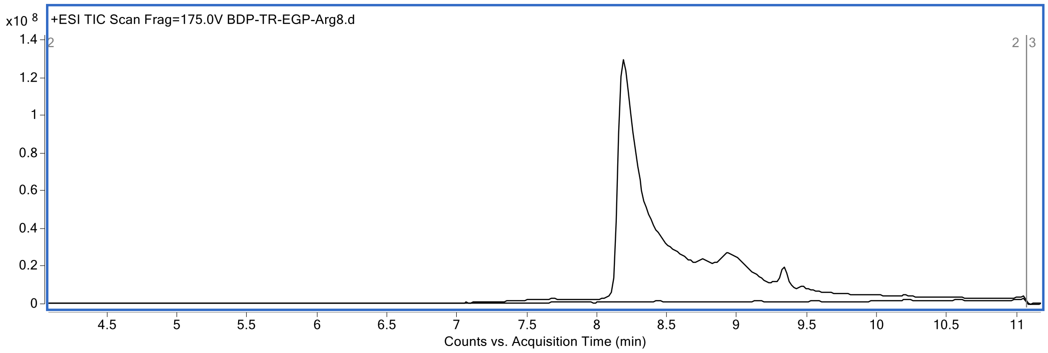

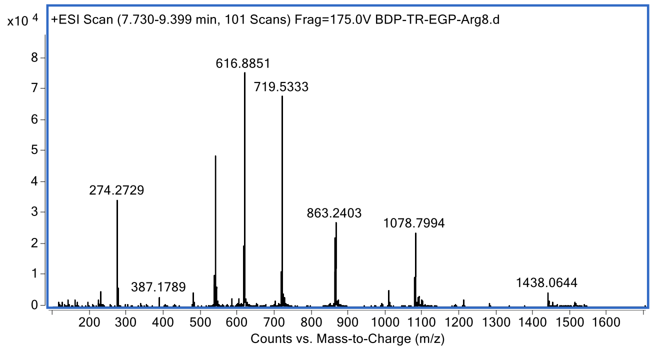

BDP-TR-gp100-Bpep

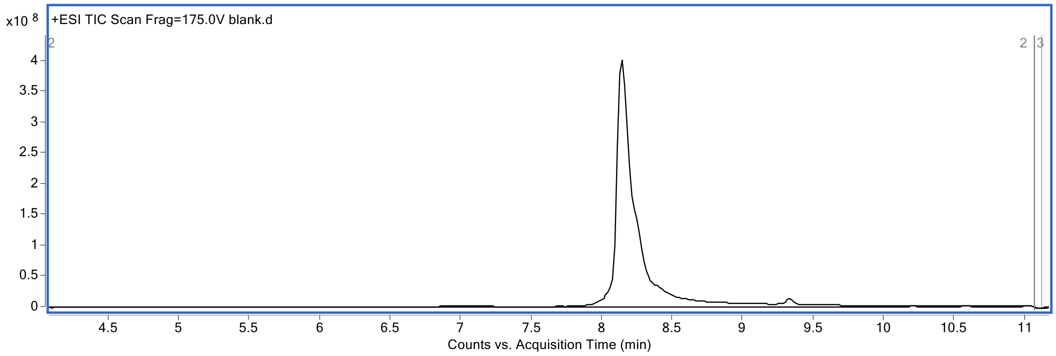

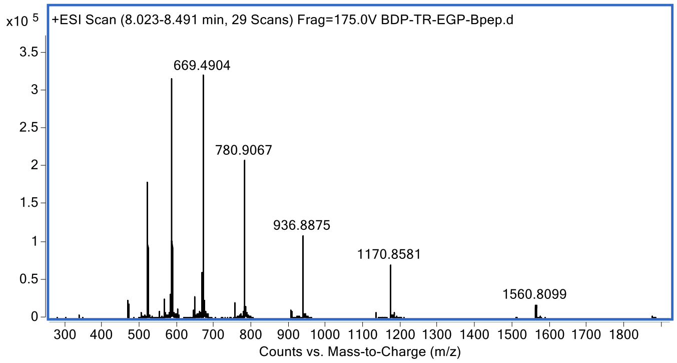

BDP-TR-gp100-MPG

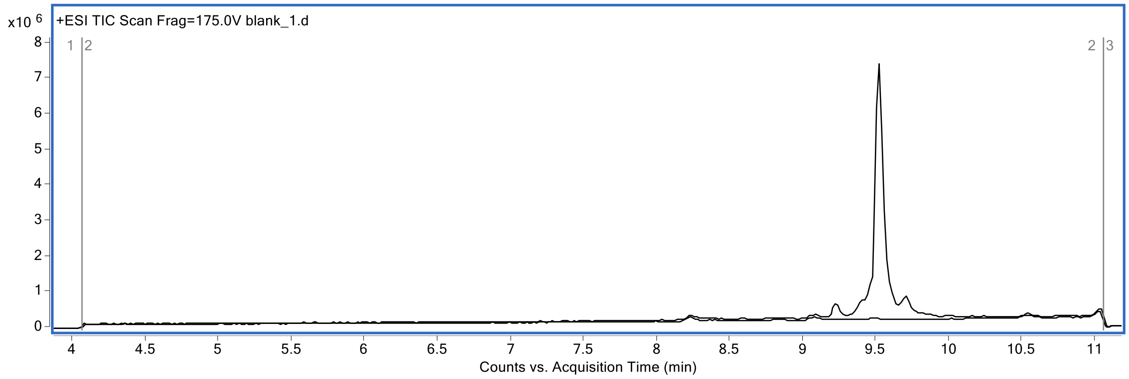

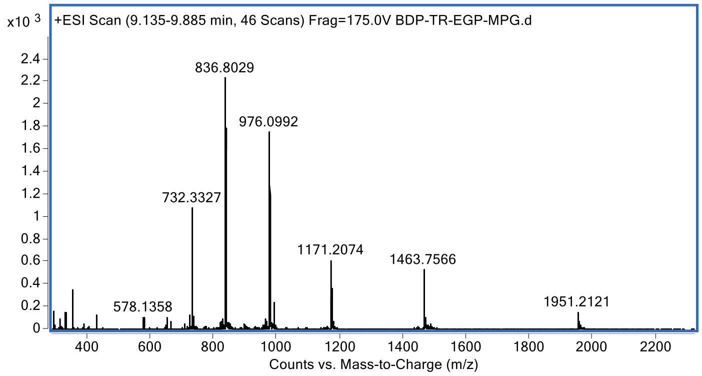

BDP-TR-gp100-pAntp

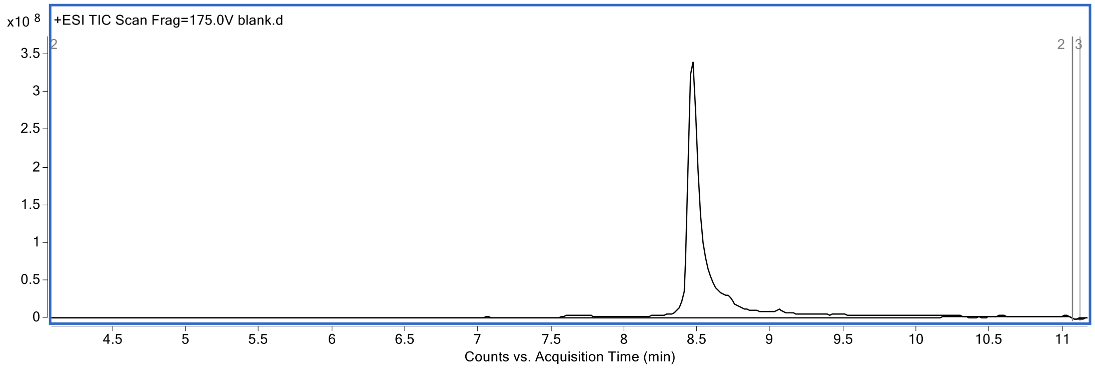

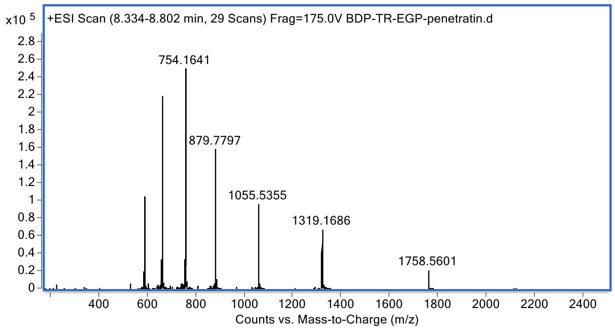

BDP-TR-gp100-TAT

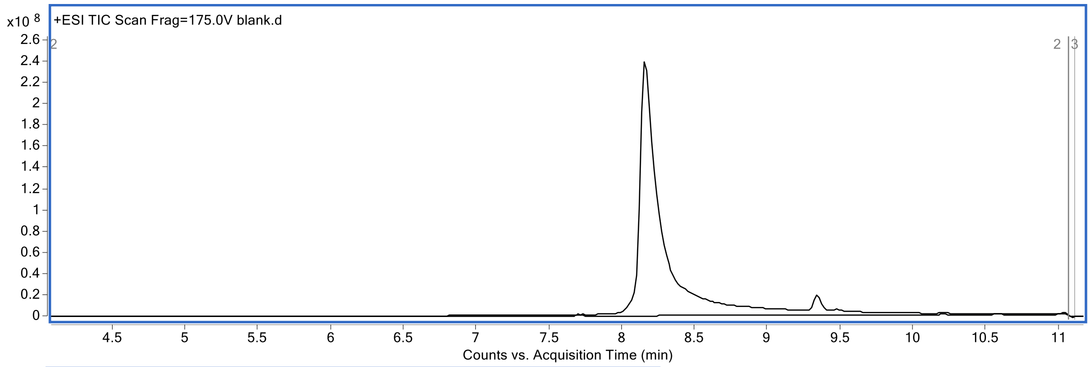

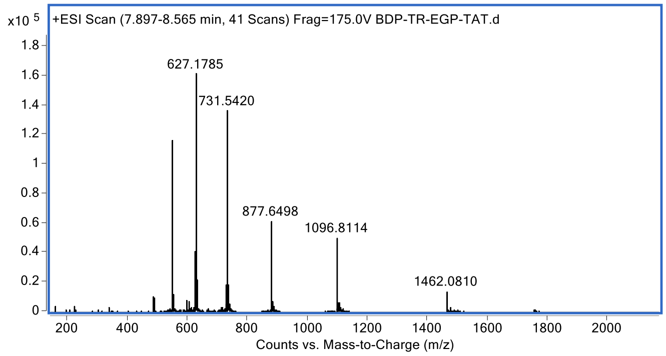

BDP-TR-gp100-TP10

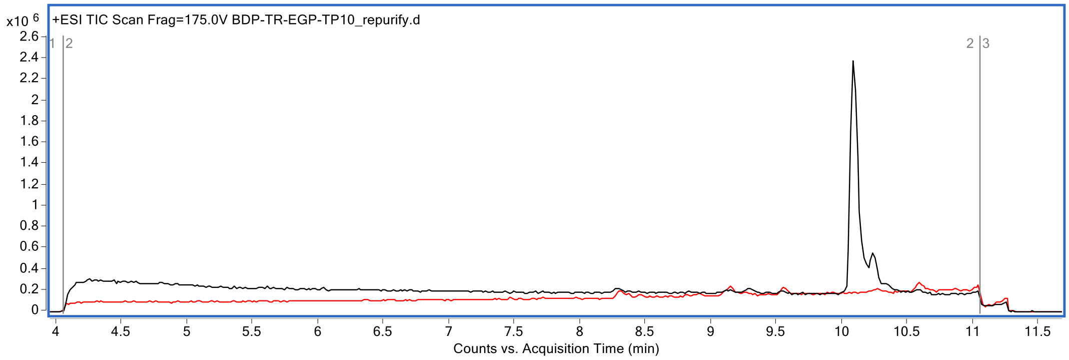

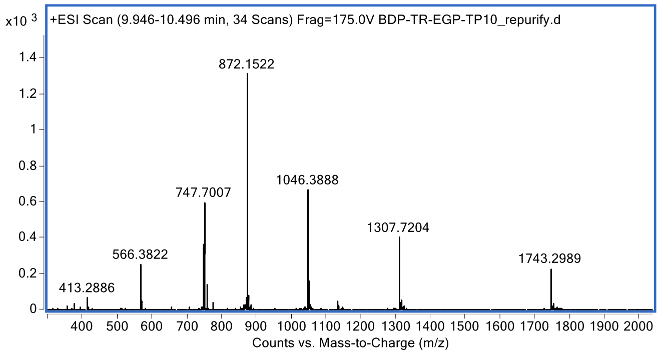

SulfoCy5-gp100 long

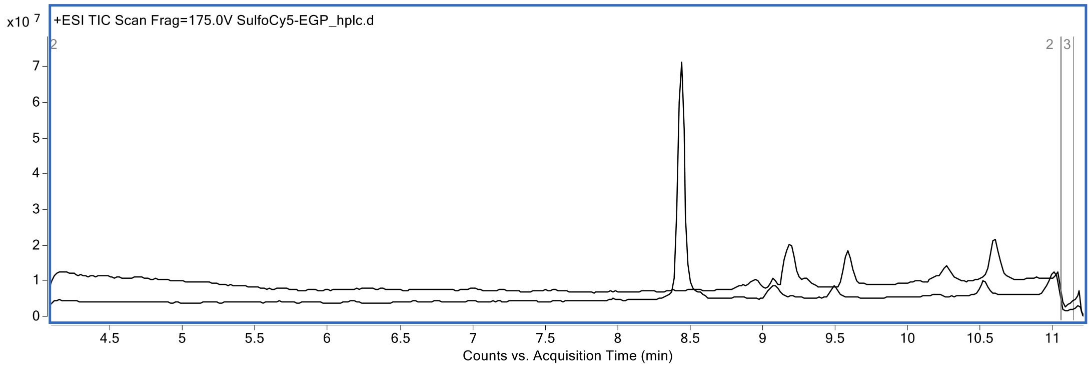

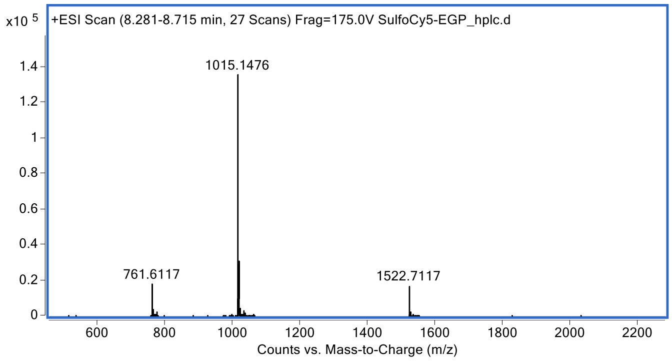

SulfoCy5-gp100-pAntp

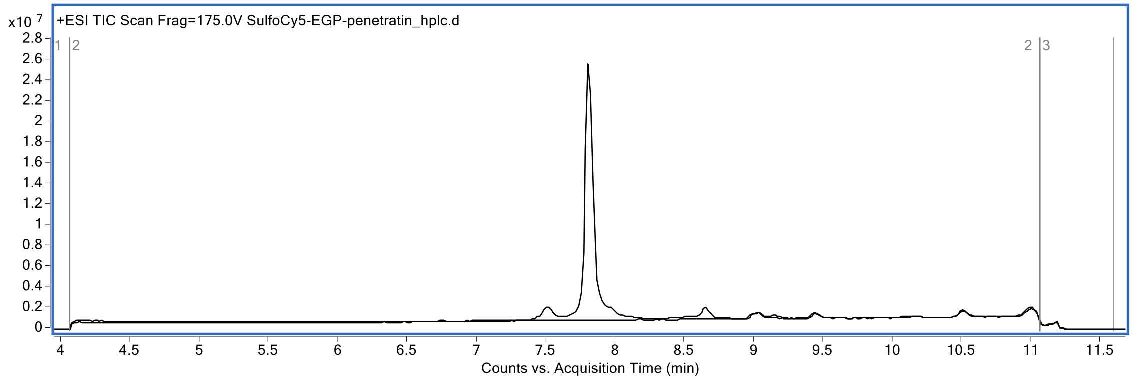

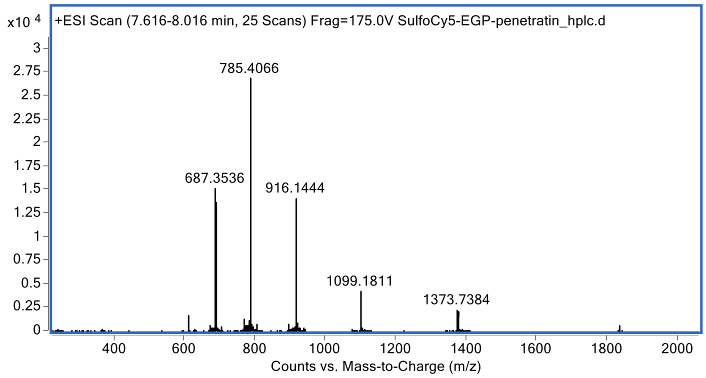

Supplement: Supplementary File [file pnas.2204078119.sapp.pdf]
